# Distribution and Molecular Evolution of *Bacillus anthracis* Genotypes in Namibia

**DOI:** 10.1371/journal.pntd.0001534

**Published:** 2012-03-06

**Authors:** Wolfgang Beyer, Steve Bellan, Gisela Eberle, Holly H. Ganz, Wayne M. Getz, Renate Haumacher, Karen A. Hilss, Werner Kilian, Judith Lazak, Wendy C. Turner, Peter C. B. Turnbull

**Affiliations:** 1 University of Hohenheim, Institute of Environmental and Animal Hygiene, Stuttgart, Germany; 2 Department Environmental Science Policy and Management, University of California, Berkeley, California, United States of America; 3 Central Veterinary Laboratory, Ministry of Agriculture, Water and Forestry, Windhoek, Namibia; 4 School of Mathematical Sciences, University of KwaZulu-Natal, Durban, South Africa; 5 Etosha Ecological Institute, Ministry of Environment and Tourism, Okaukuejo, Namibia; 6 Salisbury, United Kingdom; Yale School of Public Health, United States of America

## Abstract

The recent development of genetic markers for *Bacillus anthracis* has made it possible to monitor the spread and distribution of this pathogen during and between anthrax outbreaks. In Namibia, anthrax outbreaks occur annually in the Etosha National Park (ENP) and on private game and livestock farms. We genotyped 384 *B. anthracis* isolates collected between 1983–2010 to identify the possible epidemiological correlations of anthrax outbreaks within and outside the ENP and to analyze genetic relationships between isolates from domestic and wild animals. The isolates came from 20 animal species and from the environment and were genotyped using a 31-marker multi-locus-VNTR-analysis (MLVA) and, in part, by twelve single nucleotide polymorphism (SNP) markers and four single nucleotide repeat (SNR) markers. A total of 37 genotypes (GT) were identified by MLVA, belonging to four SNP-groups. All GTs belonged to the A-branch in the cluster- and SNP-analyses. Thirteen GTs were found only outside the ENP, 18 only within the ENP and 6 both inside and outside. Genetic distances between isolates increased with increasing time between isolations. However, genetic distance between isolates at the beginning and end of the study period was relatively small, indicating that while the majority of GTs were only found sporadically, three genetically close GTs, accounting for more than four fifths of all the ENP isolates, appeared dominant throughout the study period. Genetic distances among isolates were significantly greater for isolates from different host species, but this effect was small, suggesting that while species-specific ecological factors may affect exposure processes, transmission cycles in different host species are still highly interrelated. The MLVA data were further used to establish a model of the probable evolution of GTs within the endemic region of the ENP. SNR-analysis was helpful in correlating an isolate with its source but did not elucidate epidemiological relationships.

## Author Summary

Anthrax, the disease caused by *Bacillus anthracis*, is a neglected zoonotic diseases in the context of its impact on poor rural and periurban communities in Africa and other less developed areas of the world. Several regions of Namibia, the Etosha National Park in particular, are well known as being endemic areas for anthrax and, together, provide a good model for the investigation of the genetic diversity of *B. anthracis* circulating in livestock, wildlife and humans, and surrounding environments. The application of modern molecular strain typing techniques to the analysis of genotypic diversity, as it relates to the spatial and temporal distribution of *B. anthracis* strains in Namibia, is described in this paper. In particular, we demonstrate how it is possible to distinguish outbreaks of the disease caused by different strains from those caused by the spread of a single strain, to trace an outbreak strain back to its possible origin, and to track the routes of transmission of an outbreak strain within and between animal populations. The data described are relevant to all those concerned with monitoring, surveillance and prevention of the spread of anthrax in endemic areas.

## Introduction

Anthrax is an endemic disease in Namibia. Outbreaks occur year round in the Etosha National Park (ENP) with strong peaks in the late wet season (March and April) among zebra (*Equus quagga*), springbok (*Antidorcas marsupialis*) and wildebeest (*Connochaetes taurinus*) and in the late dry/early wet season (October peak) in the case of elephants (*Loxodonta africana*). Sporadic outbreaks also occur on private game farms and in livestock throughout the country. The earliest mortality records from the ENP are those of Ebedes [1]. Bacteriologically-based studies begun in 1983 [2], [3], [4], [5], [6] and provided insight into the temporal and spatial distribution of the disease in the Park as well as the relative roles of living and non-living vectors. Nevertheless, the lack of appropriate methods to track strains associated with particular outbreaks of anthrax and thereby resolve the overall endemic occurrence of the disease in the Park or elsewhere into a preponderance of particular strains.

The development of genetic markers for *Bacillus anthracis* over the past decade has made possible the way to a highly informative approach for monitoring the potential spread and distribution of outbreak strains of *B. anthracis*. Various sets of repeat sequences with high mutability became available for fingerprinting protocols after 2000. With respect to studies on the epidemiology of anthrax in animals, the first version of a multi loci variable number of tandem repeats analysis (MLVA) using 8 markers [7] was applied to the analysis of outbreak strains in the Kruger National Park, South Africa [8]. The discriminatory power of the method was later increased by the addition of further markers by Lista et al. [9] and van Ert et al. [10].

In 2004, Keim et al. [11] described a fingerprinting system for *B. anthracis* called PHRANA (Progessive Hierarchical Resolving Assays using Nucleic Acids) that can be used to analyze the temporal and spatial parameters of anthrax outbreaks. In this system the analysis of canonical SNPs (Single Nucleotide Polymorphisms) is used to establish phylogenetic groups, which is followed by genotyping with MLVA and finally single nucleotide repeat (SNR) analysis [12], [13] to distinguish between outbreak strains. In the study presented here a 31-marker MLVA together with the other two fingerprinting methods were used to analyze genetic relationships within a large collection of isolates from wild and domestic animals in Namibia and to identify the possible epidemiological correlations of anthrax outbreaks within and outside the ENP between 1983 and 2010. The MLVA data were further used to establish a model of the probable evolution of GTs within the endemic region of the Etosha National Park.

## Materials and Methods

### Collection of samples

Most isolates included in the study were collected after 2005, associated with an anthrax study in the ENP that began in 2006. Samples and cultures available from before that were included for the additional information they supply, albeit recognizing the limitations resulting from the small sample size. Most of the isolates originating from elsewhere in Namibia were collected at the Central Veterinary Laboratory (CVL), Windhoek, and were recovered from diagnostic samples. The collecting period spans from 1983 until 2010, with gaps in 1984–1986, 1990, 1993, 1996–1999, and 2001 and fewer than 5 isolates in each of the years 1994, 1995, 1998, 2000, and 2003.

Samples used for the isolation of *B. anthracis* consisted of either clinical swabs taken from the blood of fresh carcasses or various tissue specimens taken from older carcasses. In some cases, soil samples taken from soil underneath a carcass, heavily contaminated with blood or spilled body fluids were used. Environmental samples were either taken from 10–20 cm beneath the surface in case of dry soils by means of core samplers or from sediments of water holes using a special scoop. Swab samples with fresh blood were left exposed to the air in a biosafety cabinet for two days to ensure sporulation.

Isolation of *B. anthracis* from the specimens using semi-selective media and confirmation of diagnosis by classical bacteriological and PCR techniques, targeting both the virulence plasmids and a chromosomal marker, were performed as described in WHO [14], Beyer et al. [15], and Beyer [16].

The DNA from isolates made between 1983–1987 was kindly provided by Dr Chung Marston, CDC, Atlanta, USA.

### MLVA with 31 markers

DNA for PCR was prepared either by boiling in phosphate buffered saline (PBS) a suspension of colony material or using a commercially available kit for extraction of genomic DNA. In the first case, a 20 µl loop of a colony grown over night on blood agar was suspended in 200 µl PBS in a 0.5 µl eppendorf tube. The suspension was overlaid with 2 drops of paraffin and boiled for 15 min in a thermoblock, held at 100°C. After centrifugation at 13 000× g for 5 min at room temperature the supernatant was used for PCR. The DNAeasy Plant Kit (Quiagen) was used if a higher quality of DNA was desired.

Thirty-one loci were used from previous studies [7], [10], with the following modifications in primers used: bams21 forward (TGTAGTCCAGATTTGTCTTCTGTA), bams30 reverse (GCATAATCACCTACAACACCTGGTA), and CG3 forward (TGTCGTTTTACTTCTCTCTCCATTAC). All other primer sequences were as published. To perform a 31-marker MLVA, seven multiplex PCR reactions (primer mixes) were set up.

For multiplex PCRs 1–4 the following mixtures were used:

0.25 µM of each dNTP, 1/10 the total volume of buffer and 5 U (multiplex reaction 1) or 2.5 U (multiplex reactions 2, 3, and 4) of HotMaster TM Taq DNA polymerase (both from 5-Prime GmbH, delivered by VWR Int. GmbH, Germany), 20 µl of primer mix 1 (multiplex reactions 1, 3, and 4) or 10 µl of primer mix (multiplex reactions 2), 5 µl of DNA solution, and deionized water to a final volume of 40 µl. The Multiplex-PCRs 1, 2, 3, and 4 were carried out with the following profile: denaturation at 94°C, 2 min; 35 cycles with denaturation at 95°C, 20 s; annealing at 60°C, 30 s; elongation at 65°C, 2 min; a final elongation step at 65°C, 5 min; and cooling at 8°C. Following the PCR, the samples were purified with a commercial PCR product purification kit (Roche Diagnostics, Mannheim, Germany). The purified PCR products were diluted at 1∶30 (multiplex-PCR 1 and 3), 1∶10 (multiplex-PCR 2), and 1.20 (multiplex-PCR 4).

For multiplex PCRs 5–7 the following mixtures were used:

Each dNTP at 0.25 µM, 1/10 the total volume of buffer and 1 U of HotMaster TM Taq DNA polymerase, 15 µl of primer mix 5 or 10 µl of primer mix 6 or 7, 5 µl of DNA solution, and deionized water to a final volume of 50 µl.

The Multiplex-PCRs 5 and 6 were carried out with the following profile:

Denaturation at 94°C, 2 min; 29 cycles with denaturation at 94°C, 20 sec; annealing at 50°C, 20 s; elongation at 65°C, 45 s; a final elongation step at 65°C, 5 min, and cooling at 8°C. In the multiplex PCR 7 an annealing step at of 60°C for 30 s was applied. The 3 PCR reactions were diluted by 1∶20 (multiplex PCR 5), 1∶27 (multiplex PCR 6), and 1∶5 (multiplex PCR 7), 20 µl of each reaction was combined and 50 µl of this mix was purified by the purification kit mentioned above.

The capillary electrophoresis was performed using an ABI PRISM 310™ Genetic Analyzer (Applied Biosystems). From the multiplex reaction 1, 3, and 5 to 7 two microliter of the sample was mixed with 2 µl of a 1∶30 dilution of the size standard MegaBACE™ ET (GE Healthcare, Germany) and 18 µl of HiDi™ formamide (Appl. Biosystems). From multiplex reaction 2 and 4, a two microliter sample was mixed with 1 µl of a 1∶2 dilution of the size standard MapMarker® 1000 (BioVentures, TN, USA) and 19 µl HiDi™ formamide. The samples were boiled for 5 min at 95°C and then analyzed in a 45 min run in the ABI PRISM 310™ Genetic Analyzer (Applied Biosystems) according to the recommendations of the manufacturer. The data were analyzed with GeneMapper™ software (Applied Biosystems).

The raw data of fragment lengths were normalized by codes, reflecting the actual copy numbers of the repeat sequences where possible (Table S1). For the purpose of orientation the appropriate copy code numbers are added for the Ames ancestor strain as deduced from the sequence available at Genbank, accession No.: AE017334.2, GI:50082967. The observed fragment lengths for all alleles found in the 329 isolates of this analysis is provided in Table S4. This table also shows the correlations between the observed and expected fragment lengths. The latter are taken from the values of alleles provided by Lista et al. [9] and deduced from sequences of *B. anthracis* available on Genbank, accession No. AE017225.1 (strain Sterne), AE017334.2 (strain Ames ancestor), AE017336.2 (plasmid pXO1 of Ames ancestor), and AE017335.3 (plasmid pXO2 of Ames ancestor). Values of alleles not published were artificially added by interpolation using a repeat length as provided in Table S2.

### SNP analysis

DNA for SNP analysis was prepared as described for the MLVA.The canonical SNP analysis with 13 markers was performed as described by van Ert et al. [10] with the following modifications. Primers and probes for SNP A.Br.004 were changed to A.Br.004v2 (J. Beaudry, NAU, pers. com.) as follows: Primer A.Br.004v2 for (GCATTTGCAAGAACGCTAATG), primer A.Br.004v2 rev (GGGTCTAAGCCGATTGTAGGT), probe 6-FAM-CCAATCATGGTACTAGAT and probe VIC-ACCAATCATTGTACTAGAT. For SNPs A.Br.003 and B.Br.004 the cycling parameters on the StepOne real-time PCR system of Applied Biosystems were 50°C for 2 min, 95°C for 10 min, followed by 45 cycles of 95°C, 15 s; 90°C, 30 s; and 72°C, 30 s, followed by a final step at 60°C for 30 s.

### SNR analysis

DNA for SNR analysis was done as described for the MLVA. The SNR analysis with the 4 markers CL10, CL12, CL33, and CL35 was performed by first amplifying a larger DNA fragment, each including the original PCR-fragment as published by Kenefic et al. [12].

For amplification the following primers were used: CL10for (CCAAATGAGACCAGCAACAG), CL10rev (AGCAGGAGTGGACAGAAAAG), CL12for (CTATGGAGTTGCTCACGTTG), CL12rev (TCTCTTATACCCGCATACCC), CL33for (CATCGAATCCCTTTATCTAATTCAGG), CL33rev (GTTATACAGAGAAAAAGCGGACAT), CL35for (CGTATTGTGTTGAGAAACTTGTTG), and CL35rev (GTCGAATGCAAAGTATTCATCGT).

The cycling parameters for all amplifications were 94°C, 2 min followed by 40 cycles of 94°C, 30 s; 45°C (CL12), 50°C (CL35), or 55°C (CL10 and CL33) for 30 s; and 72°C, 30 s; followed by a final elongation at 72°C, 10 min. The PCR-fragments were sequenced by MWG Biotec AG, Germany. Sequences were analyzed using Vector NTI Advanced 10 software (Invitrogen, Germany). To determine the eventual fragment length, the primer sequences published by Kenefic et al. [12] were applied to the sequence of an amplicon and the correct fragment size was used for further analysis.

### Cluster analysis of data from MVLA and SNR analysis

Data from MLVA and SNR analysis were processed by means of the Bionumerics software package version 5.10 (Applied Maths). For cluster analysis by UPGMA a categorical coefficient was used. In the maximum spanning tree (MST) a maximum neighbor distance of 1 and a minimum number of 3 genotypes was used to build a complex.

### Determination of the relative frequency of genotypes

To determine significant differences between the relative frequency of each GT amongst isolates sampled from within the ENP we calculated simultaneous multinomial credible intervals [19] for the proportion of our isolates (pooled across all years) that were a given GT. All GTs that occurred greater than five times (GT4, GT6, GT9, G14, GT22) were considered individually and all other GTs were pooled into an “other” category. Multinomial median probabilities and 95% credible intervals were calculated in a Bayesian framework using Markov Chain Monte Carlo methods. The GT of each isolate was assumed to be drawn from a multinomial distribution with probabilities of each GT drawn from a symmetric Dirichlet prior. After a burn-in of 1000, we simulated the posterior using 100,000 samples and thinning by 10 to estimate credible intervals and median estimates. Posterior distributions were sampled for data pooled across all years as well as for individual years 2005–2010, and for years 1988–2004 grouped (due to small sample sizes).

To assess whether genetic differentiation occurred over time or between species we used the multiple regression on distance matrix (MRM) extension to the Mantel Test [20]. Briefly, genetic distances between GTs were determined using the Jaccard Index (the proportion of markers that two genotypes share). We fitted generalized additive models (GAM) of genetic distance between each pair of isolates to the temporal distance between their time of sampling and host species similarity (coded as a binary for same or different). Temporal distance and an interaction with species similarity were fitted with a smoothing function in the GAM after exploratory analysis revealed that this relationship was nonlinear. To avoid overfitting the smoothing function (as n isolates yields n*n-1 isolate pairs), we chose the smoother dimension to obtain similar estimated degrees of freedom to the mean estimated degrees of freedom obtained by generalized cross validation on random subsets of data of size equal to the number of isolates. Null distributions of all statistics of interest were obtained by fitting models to 2000 randomly permuted genetic distance matrices. Significance of terms in the GAM was determined by comparing the *F* statistic for each term to its null distribution as calculated above. Overall model fit was assessed by comparing the deviance explained by the best model to its null distribution obtained by permutation. By using the permuted models to create null distributions, the P-values reported in the results fully account for variation in number of isolates obtained during the study period. All statistical analyses were performed in ‘R’ and using the ‘*mgcv*’ package [21].

## Results

For the purposes of this paper, the term “outbreak” is defined on the basis of a “one-strain-one-outbreak” concept according to the following criteria: (i) Isolations of different GTs from infected animals are interpreted as representing different outbreaks provided that the GTs in question have been found on one or more previous occasions. Otherwise, the possibility exists that a GT may have newly emerged during the outbreak in question. (ii) Several cases of anthrax in one or more species of animals, closely related in time and space and caused by the same GT, are regarded as being part of one outbreak; when caused by different GTs, by this definition, simultaneous outbreaks are occurring. (iii) Cases caused by the same GT simultaneously but at widely separated locations, where animal movement data indicate that spread of the GT from one location to the other is highly improbable, are regarded as separate outbreaks. (iv) Cases occurring in neighbouring locations caused by the same GT but at widely separate times may represent either a recurrent outbreak or the continuation of an earlier outbreak, depending on whether monitoring has continued throughout the period concerned.

### Occurrence of genotypes and their spatial, temporal and species relations

**Figure 1:**
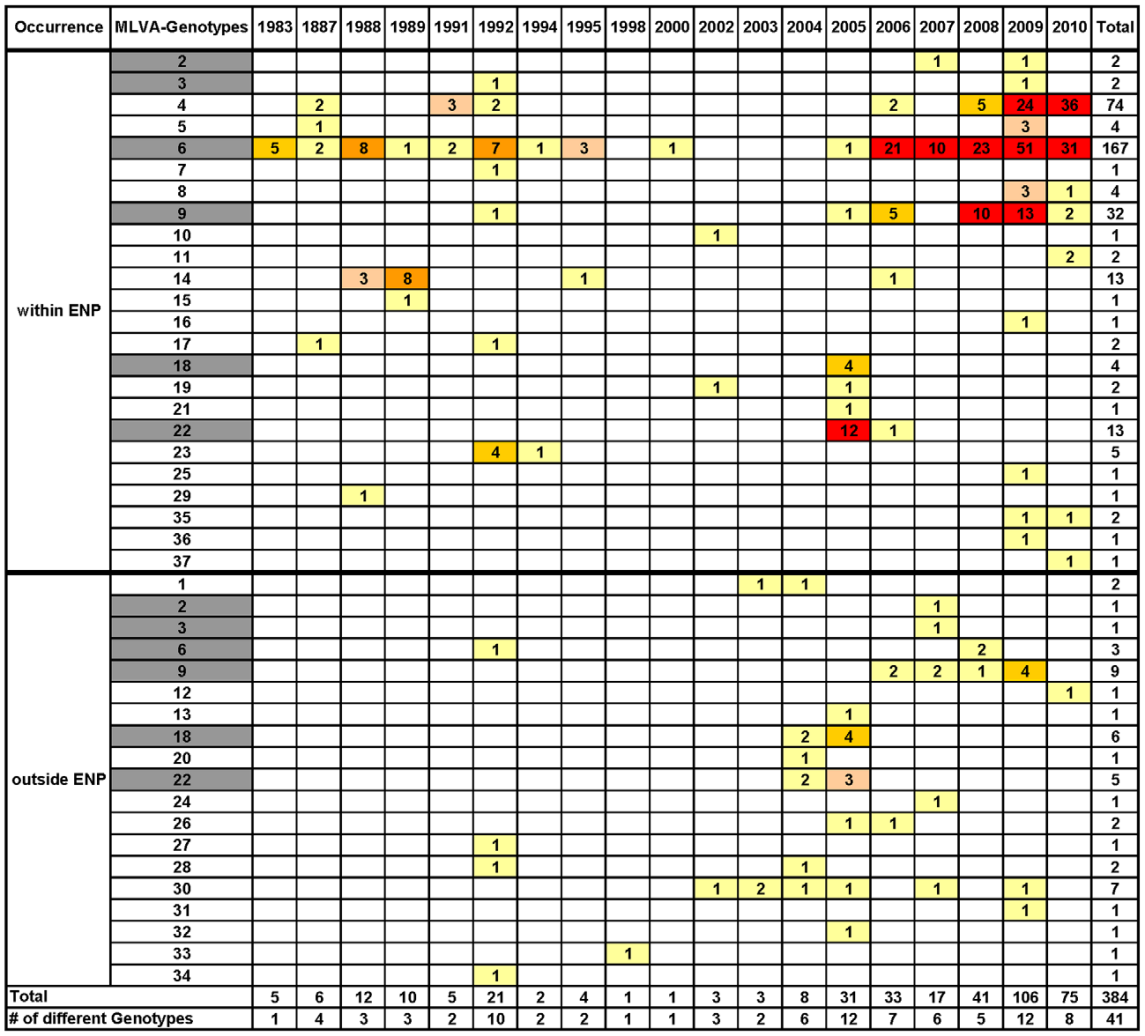
Distribution of genotypes isolated by year. The total number of isolates per year and per GT. GTs 2, 3, 6, 9, 18, and 22 (highlighted in grey) were found both within and outside the ENP.

In total, 384 isolates of *B. anthracis* from 20 animal species and 20 isolates from environmental samples in the ENP not related to a carcass were included in this analysis. Also included were seven isolates, which proved to be the Sterne vaccine strain. Six were from dead goats with unknown histories and one was an environmental isolate from a game ranch. Figure 1 and table 1 provide a summary of the distribution of isolates by GT over time and the distribution of GTs related to the species affected, respectively.

**Table 1:** Distribution of genotypes related to the species affected.

Species	Number of Isolates per MLVA-Genotype																																					Total
	1	2	3	4	5	6	7	8	9	10	11	12	13	14	15	16	17	18	19	20	21	22	23	24	25	26	27	28	29	30	31	32	33	34	35	36	37	
**Blue Wildebeest**			1	4	1	14	1		5								1					7																**34**
**Buffalo**																											1	1						1				**3**
**Cheetah**									3									1																				**4**
**Eland**																		1																				**1**
**Elephant**				3		17			2					11	1				1			2	5						1									**43**
**Hartebeest**																		1				1																**2**
**Hippo**												1																										**1**
**Kudu**									3				1									1																**5**
**Lion**										1																												**1**
**Oryx/Gemsbok**		1	1	1					3									3		1		3																**13**
**Ostrich**																						1																**1**
**Rhino**						1																																**1**
**Sitatunga**						1																																**1**
**Springbok**				5	1	34		2	6									1				2			1													**52**
**Vulture**					1	2											1																					**4**
**Zebra**		1	1	58	1	95		1	15					2		1		2			1														1		1	**180**
**Antelope**																			1																			**1**
**Cattle (Bovine)**	2																									2		1				1	1					**7**
**Goat**																						1								6								**7**
**Sheep**																								1							1							**2**
**Equine**																		1																				**1**
**Total**	**2**	**2**	**3**	**71**	**4**	**164**	**1**	**3**	**37**	**1**	**0**	**1**	**1**	**13**	**1**	**1**	**2**	**10**	**2**	**1**	**1**	**18**	**5**	**1**	**1**	**2**	**1**	**2**	**1**	**6**	**1**	**1**	**1**	**1**	**1**	**0**	**1**	**364**

A total of 37 genotypes (GT) were identified by the 31-marker MLVA; of these 13 were not found in the ENP, 18 were only found within the borders of the ENP and 6 both inside and outside the ENP. All GTs found so far in Namibia belong to cluster A, as originally defined by Keim et al. [7] (Figure S1). From the 24 GTs found in the ENP, 23 belong to SNP group 8 (A.Br.Aust94) as defined by van Ert et al. [10]. One GT (GT11) belongs to SNP group 11 (A.Br.008/009). This was the only GT not associated with any GT from a carcass and was represented by just two isolates from sediments of waterholes about 170 km apart. Two other SNP groups, group 6 (A.Br.005/006) and group 9 (A.Br.001/002) were only found outside the ENP. This is the first record of SNP groups 6, 9, and 11 in southern Africa.

**Figure 2:**
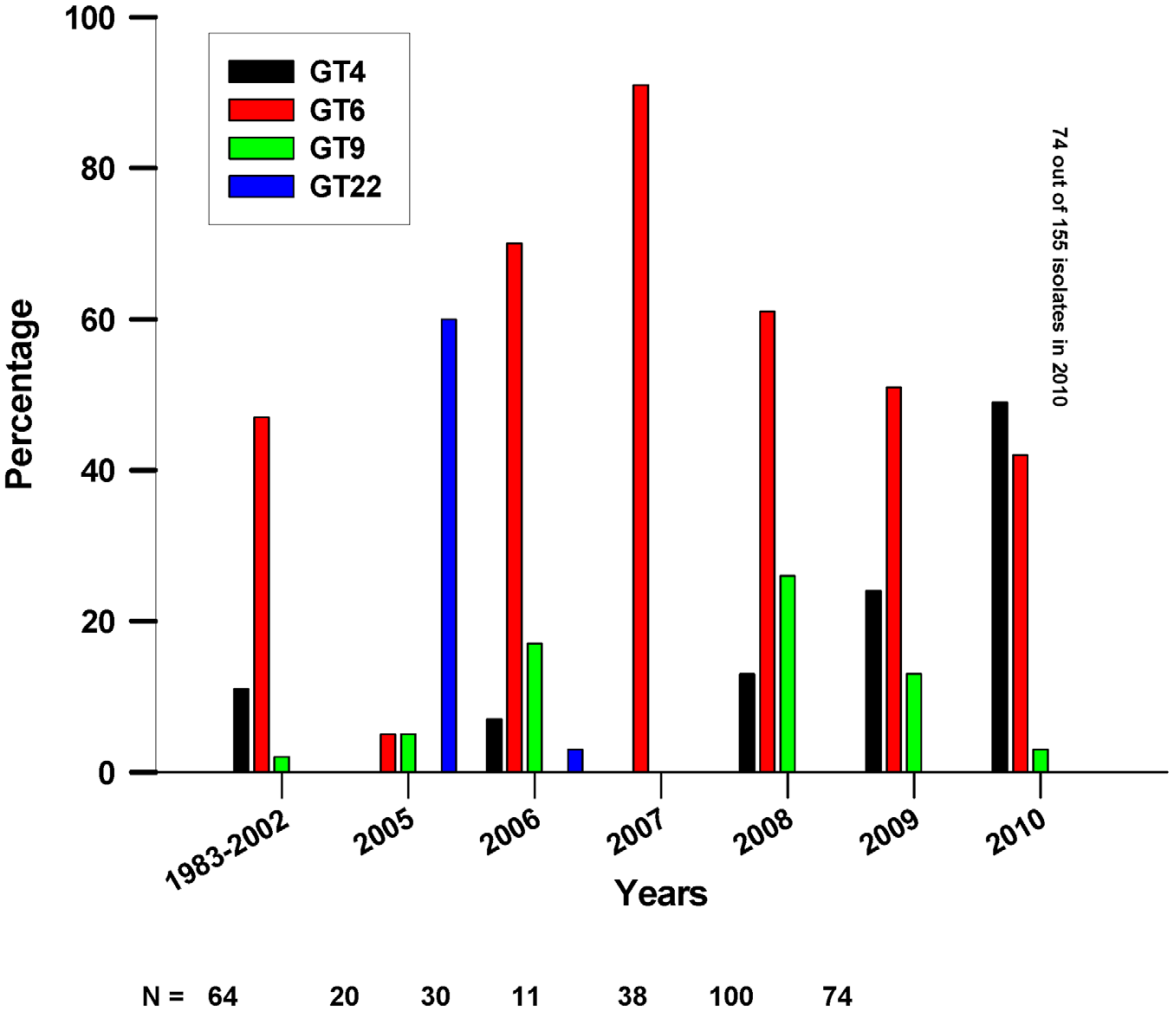
Percentage of isolates of the most prevalent GTs in the ENP per year. Numbers are given for years 2005–2010 when most of the samples were collected. Years 1983, 1987 and 1988–2002, including sampling gaps in 1990, 1993, 1996–99, and 2001, are pooled into the first data point. Total sample sizes are shown below x-axis.

**Figure 3:**
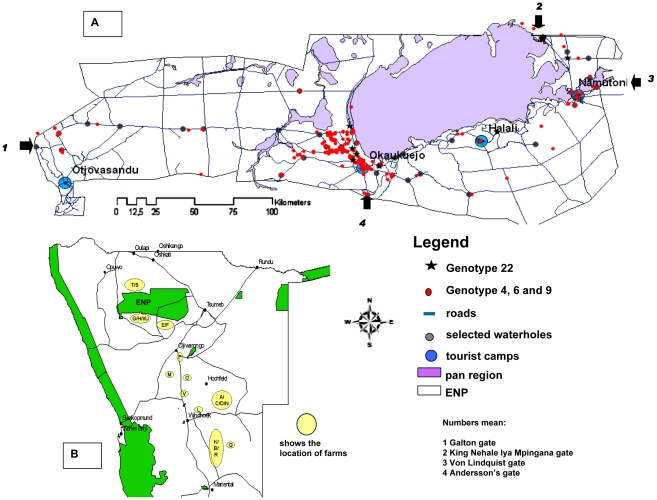
Maps of the Etosha National Park and general location of farms. (A): Main roads, pans, camps and main gates of the ENP are shown. Locations where GTs 4, 6, or 9, and 22 were found are indicated by colored marks. GTs 4, 6, and 9 were given the same label to indicate they are probably the same outbreak strain. Green areas in (B) represent National Park areas.

The temporal and spatial occurrences of the most prevalent GTs within the ENP (GTs 4,6,9, and 22) are shown in Figures 2 and 3. Additionally, the cluster analysis of all isolates in Figure S1 provides information on the spatial and species origin of each isolate by time of sampling. The 24 GTs found in the ENP are distinguishable by the apparent part they play in the epidemiology of anthrax within the Park. The majority of the GTs were only found sporadically and, apart from GT22, with just one or very few representative isolates (Figure 1). While many GTs were found once or a few times only at the beginning or at the end of the study period, some GTs were found both once in the early years and then again many years later on only single or a few occasions. GT22 was unusual in being the cause of a substantial outbreak affecting several species and being the most prominent GT in 2005 (Figure 2 and Table S3). The proportion of isolates sampled from 2005 that were GT22 was greater than that of any other genotype for that year, as calculated by simultaneous multinomial 95% credible intervals [19] (Figure S2). It was isolated only once more in 2006.

**Figure 4:**
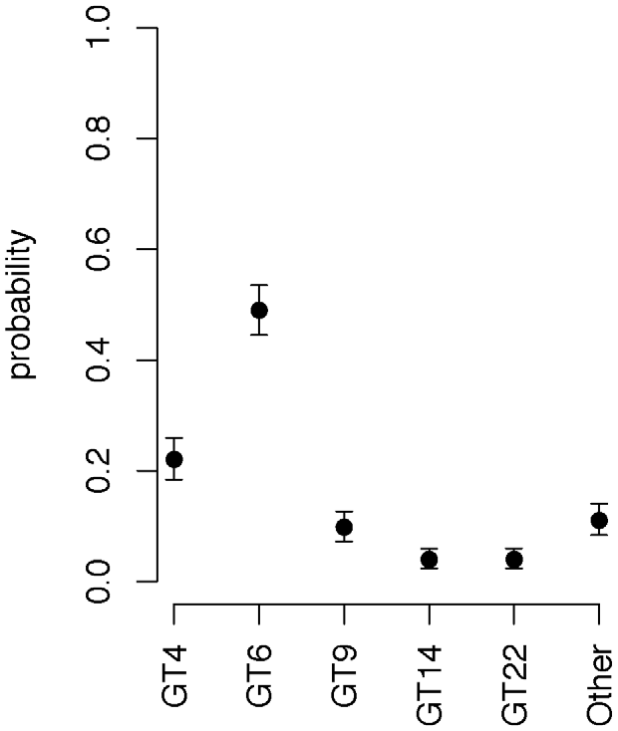
Median probabilities and 95% credible intervals for genotypes sampled from the Etosha National Park. GTs occurring ≤5 times are pooled into the “Other” category.

In contrast, GT4, GT6 and GT9 were found more frequently. GT6 was first found from samples of dead elephants and zebras in 1983–1988 and then again most years after that in which cultures were available. This GT accounted for 167 (49.5%) of the 337 isolates in the ENP (Figure 1) and was found throughout the Park, from mountain regions near the western border to the plains in the northeast, spanning 300 km (Figure 3). GT4 and GT9 were first found in 1987 and 1992 and represent 74 (22%) and 32 (9.5%) of the 337 isolates from the ENP, respectively. GTs 4, 6, and 9 were each significantly more prevalent than all other isolates when all years are pooled (Figure 4). Together the three GTs account for more than 80% of all isolates from the ENP.

**Figure 5:**
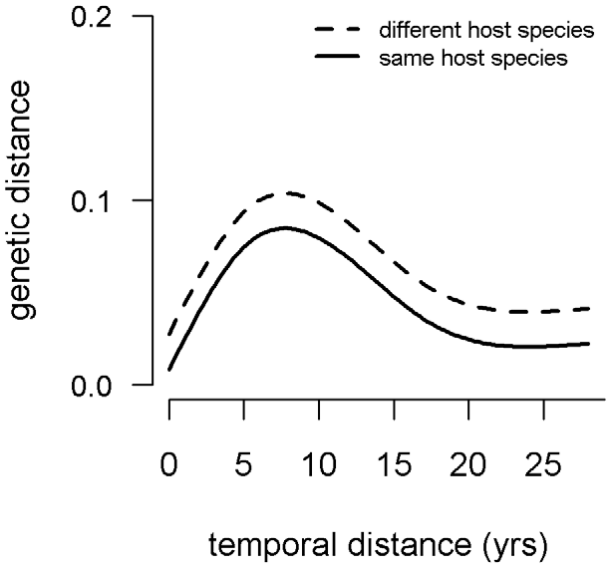
Genetic distance as a function of temporal distance and host species similarity. Isolates of *Bacillus anthracis* collected from the ENP during the period 1983–2010 are included.

The presence of a dominant outbreak clone in the park was further tested by evaluating the relation between genetic distances between each pair of isolates and the temporal distance between their times of isolation as well as whether they were from the same host species or not. The best fit generalized additive model (GAM) performed in a multiple regression on distance matrices framework (MRM) included both a nonlinear smoother function of temporal distance (*p*<0.0005) and a term for species similarity (*p*<0.0005). The interaction between the terms was not significant (*p* = 0.61) and was removed from the final model. The temporal relationship is characterized by increasing genetic distance with temporal distance to a peak at around 8 years apart and then decreasing genetic distance thereafter (Figure 5 and Figure S3). This model explained a significant proportion of the deviance (0.149, *p*<0.0005). The average proportion of markers differing between isolates was 0.031 for isolates from the same host species and 0.054 for those from different species.

**Figure 6:**
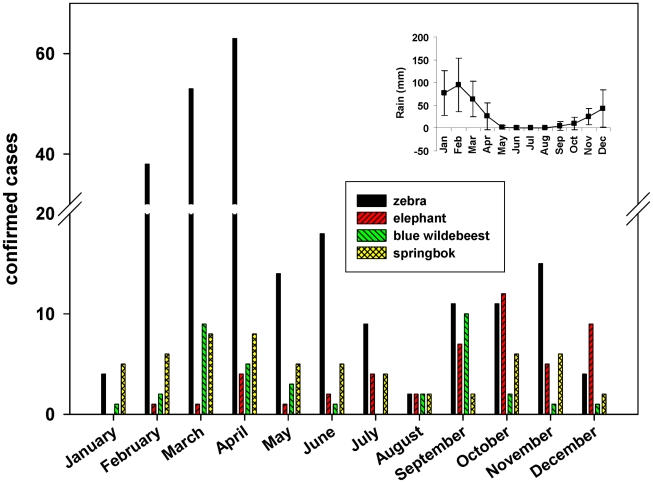
Confirmed cases of anthrax in the four most commonly affected species in the ENP. The period between 1988–2010 is shown. Isolates prior to 1988 have been excluded from this figure, having been collected during only short field studies in the Park. The insert indicates the mean monthly rain fall (in l/m^2^) in the Okaukuejo region, in 1983–2010.

The distribution of cases monitored in this study supports the concept of persisting or recurring outbreaks at least in the case of zebra and springbok, with peaks in cases occurring during the late wet season. Also elephants may contract the disease any time of a year but are more likely to do so in the late dry/early wet season (Figure 6). By categorizing the isolates by GT and time of isolation (Table S3) the overall endemic occurrence of anthrax in the ENP can be differentiated into long lasting or recurrent outbreaks caused by the same GT and short term sporadic outbreaks caused by numerous different GTs. Long lasting outbreaks may continue for more than one year, such as those caused by GT6 in 2007 and in 2009. Those caused by GT6 in 1992, 2006, and 2008, by GTs 4 and 9 in 2008 and by GT9 in 2009, may exemplify recurring outbreaks. There are many examples of separate outbreaks occurring simultaneously in the same region, such as those caused by GT18 and GT22 in 2005, by GTs 4, 6, 9, 14, and 22 in 2006, and by GTs 4, 6, and 9 in 2008 and 2009, all in the Okaukuejo region of central ENP.

### Probable evolution of GTs of *B. anthracis* in the ENP

**Figure 7:**
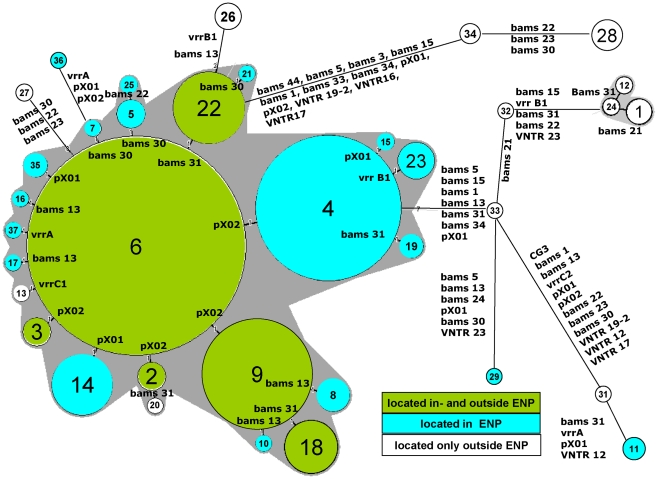
Minimum spanning tree of 377 Namibian isolates typed by MLVA. Clustering of MLVA profiles was done using a categorical coefficient. The MLVA-GTs are displayed as circles with the appropriate GT number. The size of each circle symbolizes the number of isolates of this particular GT. GTs differing in only one marker are combined in a complex seen as a gray halo if at least 3 GTs fulfill this criterion. The marker(s) on which the mutation is located is shown. Distances between circles do not reflect the correct phylogenetic distances. The vaccine isolates are not included. The relations within this tree remain the same if an outgroup like the B-cluster (as used in Figure S1) is included (not shown).

The close temporal and regional relationship between GT4, GT6, and GT9 prompted an interest in the evolutionary distances between these and the other GTs found. For this purpose all GTs, except GT30 from the vaccine strain, were subjected to minimum spanning tree (MST) analysis, which indicated that GT6 might be the ancestor of all other GTs found in the ENP. The MST in Figure 7 shows the GTs with the markers in which they differ. GTs 4 and 9 differ from GT6 in only one highly mutable marker located on the pXO2 plasmid. Mutations at the same locus are also responsible for the possible evolution of GTs 2 and 3 from GT6. Differences in only one or two markers, located in both high and lower mutable markers, indicate the presence of a closely related clonal group of *B. anthracis* strains within the ENP. Only the GTs 11 (from sediments of two far apart waterholes), 29, and 36 are more distantly related and differ from GT6 by, respectively, 16, 9 and 4 markers.

### Relation of GTs found outside the ENP

Six GTs were found both within and outside the ENP. Among them only GT18 and GT22 were also found in livestock, though both on only one occasion. GT18 was found four times in the ENP between October and November 2005 and also that year and the year before on four game farms, A–D, all located east of Windhoek, some 400 km southeast of the ENP (Figure 3). SNR analysis was able to resolve the ten GT18 isolates into a further six SNR types. On each of farms A and B, the two isolates found were of the same SNR type but different from any other SNR type in this GT (Figure S4). GT22 was found on 13 occasions in the ENP between June 2005 and August 2006. In October 2004 this GT was isolated from both a goat (*Capra* spp.) at a game farm near the south border of the ENP (farm E) and an oryx (*Oryx gazella*) at a second farm (farm F) located a few kilometers west of farm E. These two isolates had the same SNR profile. However, three isolates from the same farm F, from a kudu (*Tragelaphus strepsiceros*) (June 2005), a hartebeest (*Alcelaphus buselaphus*) (July 2005) and an oryx (October 2005) belonged to three different SNR types within GT22 (Figure S5).

Other GTs occurring both inside and outside the ENP were GTs 2, 3, 6, and 9. GT2 has only been found recently, twice in the Okaukuejo region in 2007 and 2009 and once in 2008 on game farm G approximately 35 km south-west of Okaukuejo. GT3 was found twice in the ENP – once each in the northeast of the Park in 1992 and in the west of the Etosha Pan in 2009 – and again once outside the Park, some 450 km to the southeast of the ENP in 2007. GT6, the most prominent GT within the ENP was isolated once in 1992 from the Caprivi province and once again in 2008 from an elephant near the north-east border of the Park (Okashana). GT9 was found on 31 occasions in the ENP between 2005 and 2010. In 2006 and 2007 the same GT was found near the southern border of the ENP, killing one kudu each on nearby farms H and I and an oryx on farm G. Though being of the same GT these three isolates were of two SNR types, both different from most of the SNR types found among the ENP isolates of GT9. In 2009 GT9 caused an outbreak on two other game farms east of Okaukuejo (farms K and L) where an oryx and a number of cheetahs (*Acinonyx jubatus*) died after being fed meat from the infected oryx. The route of infection was verified by these isolates having the same but unique SNR type (Figure S6).

Thirteen GTs were only found outside the ENP. Among them GT28 was isolated both from a wild animal (buffalo [*Syncerus caffer*], in 1992) and a domestic animal (bovine in 2004), both from the Caprivi region, while GTs 12, 13, 20, and 34 were found only in game animals, and only once each. Among the latter, GT13 from farm G is closely related to GTs 6 and 9 (Figure 7) which are both also present at this farm.

There are six GTs [1], [24], [26], [31], [32], [ and 33] which were isolated only from domestic animals, namely cattle and sheep from different farms in the northern, eastern and southern parts of the country. All of them are genetically rather distantly related to GTs from within the ENP except GT26, which differed from GT22 in only two markers.

GT30, representing the locally used veterinary vaccine strain was isolated from six goat carcasses, from different farms between 2002–2009, and once from the environment (water pan) of a game ranch.

## Discussion

Of the 37 GTs found so far in Namibia, none belong to the large cluster B1 of Keim et al. [7]. GTs of the B1 cluster have so far only been isolated in southeast Africa (northeast of South Africa, southeast of Zimbabwe and an unspecified region of Mozambique) [8], [10, unpublished findings], though cluster B1 and B2 isolates are present in historical strain collections of many European countries and the USA [10], [22]–[24] and even among recent isolations from outbreaks in Germany and France (unpublished data).

Of the 24 GTs found in the ENP, 23 belong to a single SNP group (A.Br.Aust94) as defined by van Ert et al. [10], supporting the theory of a clonal expansion of an ancestor strain, introduced to the region a long time ago. The only exception are two isolates of GT11, belonging to SNP group 11 differing from GT6 by 16 markers. Together with GT36 and GT29, differing in 4 and 9 markers from GT6, these three GTs are highly unrelated to all other GTs present in the ENP and may, therefore, represent separate introductions of *B. anthracis* to that region. Two other SNP groups, group 6 (A.Br.005/006) and group 9 (A.Br.001/002) were only found outside the ENP. This is the first record of SNP groups 9 and 11 in southern Africa. We did not find SNP groups 7 (A.Br.003/004) and 5 (A.Br.Vollum) in Namibia, both described by van Ert et al. [10] in South Africa, nor – in concordance with the lack of any isolates of the B-branch – any SNP group of the B-branch.

While an expected increase in the number of different GTs did occur with an increase in the number of isolates collected each year in the last six years, GTs 4, 6, and 9 remained the only ‘dominant’ ones in terms of their temporal occurrence and the number of cases they caused. DNA from the earliest isolates available from the ENP [2], [3] revealed the presence of GT6 and 4 in the Park for more than 25 years. Since GTs 4 and 9 differ from GT6 by only one mutation in a highly mutable marker on the pXO2 plasmid, these three GTs can be considered one strain that is likely to have been causing outbreaks in the ENP for a very long time. All other GTs, with the exception of GT22, which was the prominent GT in 2005 have only been found sporadically. The statistical relationship between time and genetic differentiation appears counter-intuitive. The greatest genetic distance between isolate pairs occurred for isolates obtained at an intermediate distance apart in time (Figure 5). However, the decline after about 8 years apart indicates that isolates far apart in time were often closely related. This suggests that while certain GTs arose and faded away during the sample period, certain “dominant” GTs were present throughout the study period, and were particularly prevalent during the beginning and end of this period.

Genetic distance was also significantly greater on average for isolates from different host species. Given that GTs were highly related in general, we do not believe that the species-GT relationship is indicative of adaptive evolution. Rather, we suppose that these results suggest that the transmission cycles of anthrax in the ENP are somewhat differentiated by host species, owing to their ecological and behavioral differences. While significant, the effect of species dissimilarity was not extreme (Figure S3). Thus, although there are transmission processes that differ among species, the transmission cycle of *B. anthracis* in the ENP appears to be highly connected among species.

Our results raise the question of the kind of relation between the markers used for MLVA and the part a GT will play as an outbreak strain in the long term. Despite the obvious stochastic effects of sampling and genetic drift on the one hand and environmental and ecological factors affecting transmission on the other hand, the overall picture of spatial and temporal distribution of GTs presumably also depends on the genetic features of the strains present. Genes coding for superior survival in the environment, for example sporulation capability [25] and tenacity after release from an infected host and virulence features, such as lower infectious dose, higher replication rate within a host, and higher resistance against the innate immune mechanisms of a host, may give some GTs a fitness advantage over others. As far as is known, several of the VNTRs used in the 31 marker MLVA are part of coding sequences in chromosomal and plasmid genes. However, with a few exceptions, such as genes where bams13, bams30, bams24, bams34, bams44, and vrrC1 and vrrC2 are located, they would not be expected to play a major role in the mechanisms mentioned above. Thus, it remains unknown whether the three most frequently isolated GTs dominate the genotypic landscape due to fitness advantages. Further whole genome and transcriptome analysis will allow investigation of the possible genetic and phenotypic relations indicated by the different levels of occurrence of the GTs observed in this study.

Our minimum spanning tree (MST) suggests that GT6 is the ancestor of all other GTs found. The reliability of evolutionary trees mainly depends on the rate and stability of the mutations used to build the tree [10],[11],[26],[27]–[29]. For our strain collection the mean index of diversity of all 31 markers is 6.06 (from 0.0 to 59.5). The number of different alleles ranges from 1 to 11 (Table S4). All VNTR-markers used in our analysis were shown to be stable during routine bacteriological diagnosis and passage in mice and rabbits [30]. Our MST may, therefore, represent a reasonable model for the evolution of *B. anthracis* within the ENP.

If a competent vector is present, newly evolving GTs may spread rapidly throughout the Park and sometimes beyond its borders. GT22 may provide an example of a rapidly spreading outbreak strain, raising the question as to what can serve as an appropriate vector under the conditions of the ENP. Flowing water, water holes, and airborne spread of spores are rather unlikely to play a role [6], [31]. Depending on the geographical and seasonal conditions, live vectors are probably the principal spreaders of the disease over long distances either mechanically [14], or by infected animals incubating the agent while, at the same time, moving long distances. In this regard elephants, among other herd animals, may serve as spreaders of anthrax within the ENP and beyond its fenced borders. In 1988, GT6 was found in elephants simultaneously in the far west of the Park and about 300 km to the east near Namutoni. GT22 was found in October 2005 from two elephants, one in the Halali region and the other approximately 70 km away in the north-east of the Park. While currently available movement data for elephants in the ENP do not indicate these cases were located along known movement corridors, the latter two cases occurred within a feasible distance of elephant movement. GT23 was found in April 1992 in elephants near Okaukuejo and some 50 km away around Halali, in this case in regions that are on known elephant movement routes. The elephant deaths outside the Park from GT6 and GT9 in October 2008 at the same time as these GTs were being isolated from cases within the Park provides support for this hypothesis. However, this theory does not exclude the possibility of casual infections outside the Park, particularly because the occurrence of GTs 6 and 9 is not restricted to the ENP.

The relationship between those GTs found both in the ENP and outside the ENP can only be speculated upon. The ENP is a >22000 square kilometers territory, comprehensively described in numerous public websites (see http://de.wikipedia.org/wiki/Etosha-Nationalpark for an overview). While the park perimeter is fenced, animals do occasionally break through the fence (particularly elephants) or go underneath it (particularly through holes dug by warthogs). Farms located at close vicinity to the ENP may be part of the regional ecosystem promoting the spread of outbreak strains between the ENP and farms. For instance, GT13, isolated from a farm abutting the ENP is as closely related to GT6 as the majority of the GTs found within the park and, therefore, probably belongs to the same group of descendents from GT6 (Figure 6). To investigate possible epidemiological relations further, SNR analysis was applied to the MLVA clusters of GT9, 18, and 22, as single nucleotide repeats are known to have a very high mutation rate [36], [37] and, therefore, are applicable to forensic and outbreak analysis [11]. While our SNR analysis clearly correlated isolates with their source of sampling, its usefulness as a tool to prove or disprove possible transfers of outbreak strains between different locations was rather limited. In case of high numbers of SNR types within a MLVA based genotype, the analysis obscures rather than clarifies the epidemiological relationships. Isolates from the same GT but differing in their SNR type can still be considered “the same outbreak strain” if other epidemiological criteria, such as regional and temporal relationships or certainty as to how the *B. anthracis* was spread, suggest a highly likely epidemiological connection between two outbreaks, as in the case of the four different SNR types within GT22 at the same game farm. However, the same SNR type as in the case of the cheetahs dying from contaminated meat strongly supports the source and route of infection.

Thirteen of the total of 37 GTs were only found on farms in different regions of Namibia. Seven of these, including the vaccine strain, were only found in domestic animals housed at 8 different locations. Though most of the GTs isolated outside the ENP have only been found so far from single cases, the genetic distance from isolates within the Park indicates separate epidemiological anthrax cycles in livestock. There have been temporally separated but closely related cases of anthrax in livestock on nearby farms. However, assessing whether or not this also represents possible epidemiological connections between such outbreaks requires more data than are currently available.

In conclusion, the molecular characterization of isolates from outbreaks of anthrax in Namibia has permitted an understanding of the divergence of *B. anthracis* and probable evolution of an ancestral strain introduced into the region long ago, revealing substantial genetic distances between the strains circulating within the ENP and those in livestock. It has also confirmed the value of the 31-marker MLVA for revealing single outbreak events within an otherwise endemic occurrence of anthrax in animals. The routine application of molecular characterization offers a highly valuable addition to conventional epidemiological methods for the surveillance and prevention of this neglected disease.

## Supporting Information

Figure S1 **Clusteranalysis of 31-marker MLVA data on 377 Namibian isolates.** An isolate of the B-branch (Malilangwe 2005) was added as an outgroup. To protect the privacy and security of farm owners, the identities of farms are designated simply by upper case letters.
(TIF)

Figure S2 **Median probabilities and 95% credible intervals for genotypes sampled from the Etosha National Park.** GTs occurring ≤5 times are pooled into the “Other” category. Numbers are given for years 2005–2010 when most of the samples were collected. Years 1983, 1987 and 1988–2002, including sampling gaps in 1990, 1993, 1996–99 and 2001, are pooled into the first data point. The first plot for “all years” is identical to Figure 4 in the text.
(TIF)

Figure S3 **Distribution of genetic distances by host species similarity.**
(TIF)

Figure S4 **Cluster analysis of SNR data from all isolates of GT18.** To protect the privacy and security of farm owners, the identities of farms are designated simply by upper case letters.
(TIF)

Figure S5 **Cluster analysis of SNR data from all isolates of GT22.** To protect the privacy and security of farm owners, the identities of farms are designated simply by upper case letters.
(TIF)

Figure S6 **Cluster analysis of SNR data from all isolates of GT9.** To protect the privacy and security of farm owners, the identities of farms are designated simply by upper case letters.
(TIF)

Table S1 **SNP groups and code values for the 31 markers used in MLVA.** Code numbers are identical with copy numbers of the repeat sequences where possible. For the purpose of orientation the appropriate copy code numbers are added for the Ames ancestor strain as deduced from the sequence available on GenBank, accession No.: AE017334.2 GI:50082967
(XLS)

Table S2 **Observed fragment lengths and code numbers used in the 31-marker MLVA.** Fragment lengths observed on an ABI 310 capillary electrophoresis are compared with expected fragment lengths. The latter are taken from the values of alleles provided by Lista et al. [9] and deduced from sequences of *B. anthracis* available at Genbank. Values of unpublished alleles were deduced by interpolation using the given repeat lengths.
(XLS)

Table S3 **Records on ENP isolates used for outbreak analysis.**
(PDF)

Table S4 **Diversity indices and allele numbers of MLVA markers with respect to the collection investigated.**
(XLS)
