# Supplementary figures and images for: Distribution and Molecular Evolution of Bacillus anthracis Genotypes in Namibia
Source: PLoS Negl Trop Dis. 2012 Mar 6;6(3):e1534. doi: 10.1371/journal.pntd.0001534 (PMC3295808; doi:10.1371/journal.pntd.0001534)

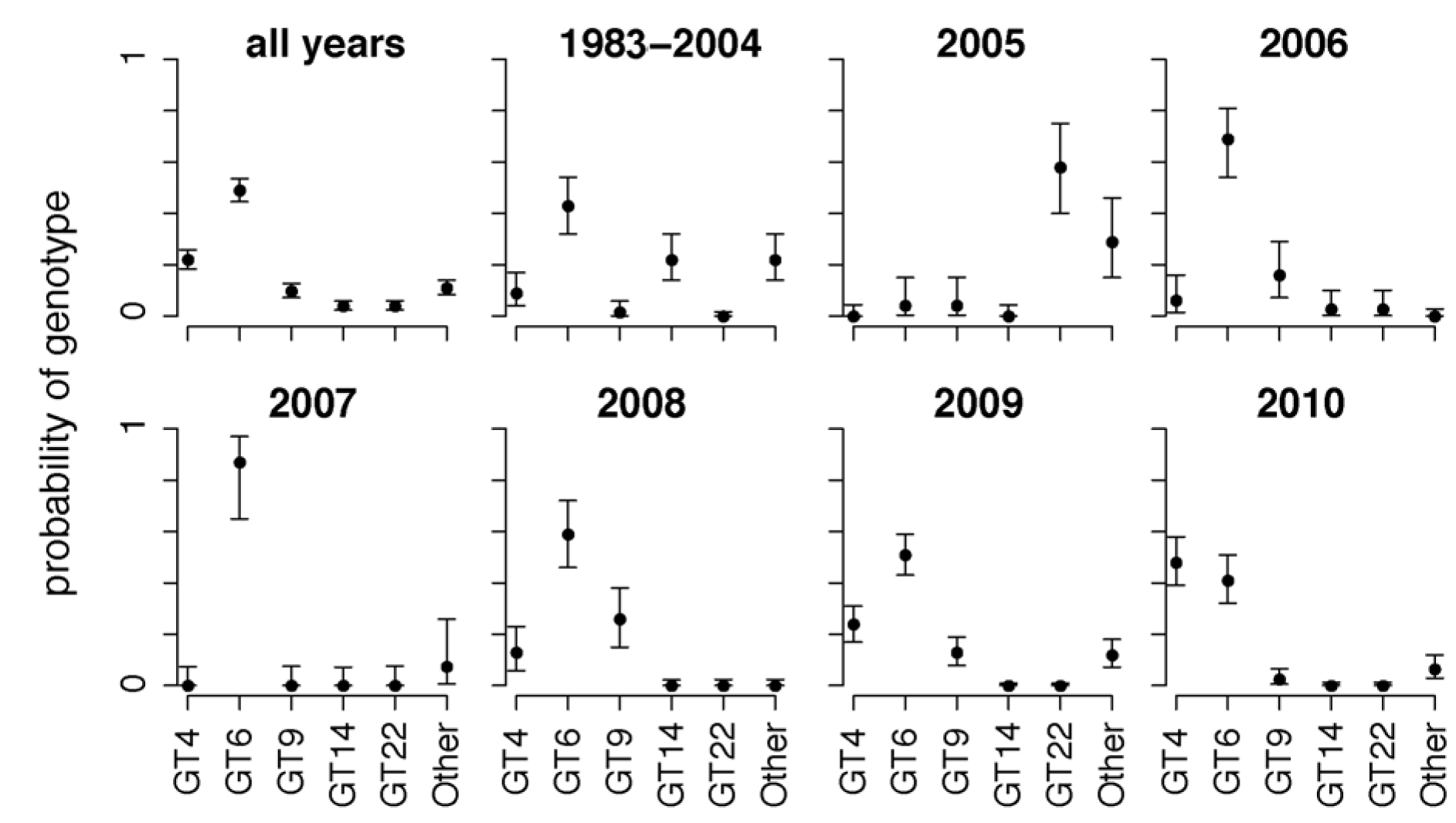

Supplement: Figure S2 — Median probabilities and 95% credible intervals for genotypes sampled from the Etosha National Park. GTs occurring ≤5 times are pooled into the “Other” category. Numbers are given for years 2005–2010 when most of the samples were collected. Years 1983, 1987 and 1988–2002, including sampling gaps in 1990, 1993, 1996–99 and 2001, are pooled into the first data point. The first plot for “all years” is identical to Figure 4 in the text. (TIF) [file pntd.0001534.s002.tif]

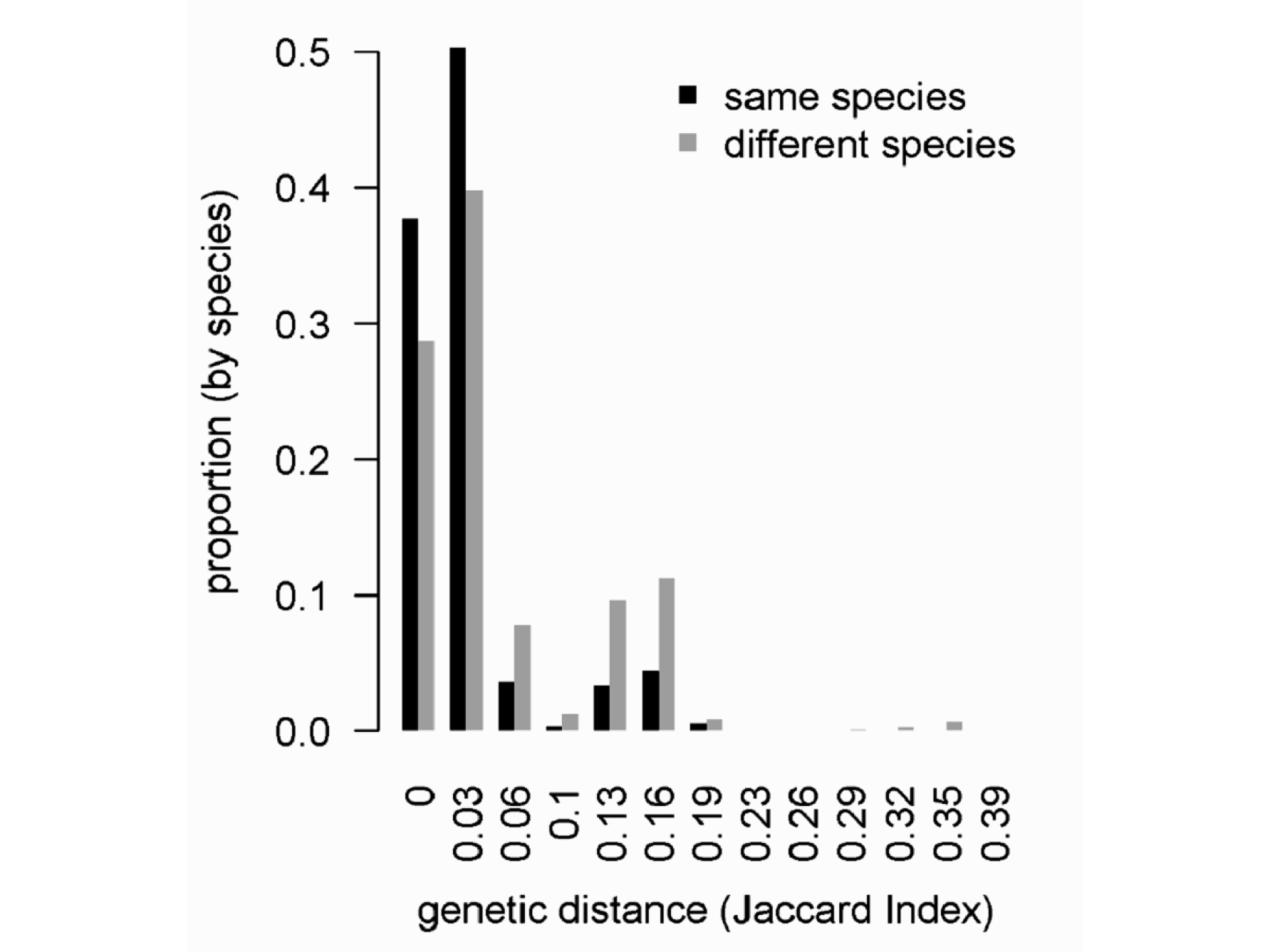

Supplement: Figure S3 — Distribution of genetic distances by host species similarity. (TIF) [file pntd.0001534.s003.tif]

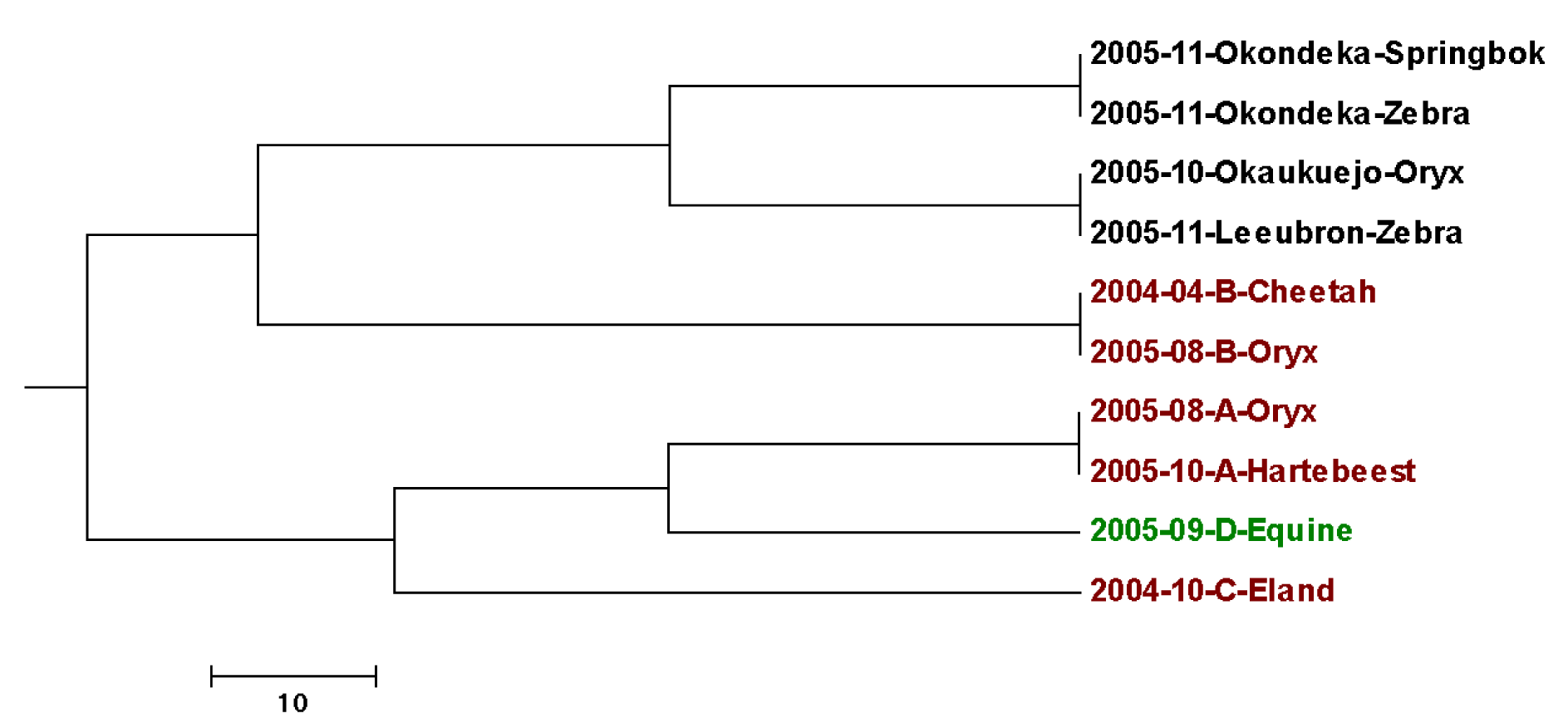

Supplement: Figure S4 — Cluster analysis of SNR data from all isolates of GT18. To protect the privacy and security of farm owners, the identities of farms are designated simply by upper case letters. (TIF) [file pntd.0001534.s004.tif]

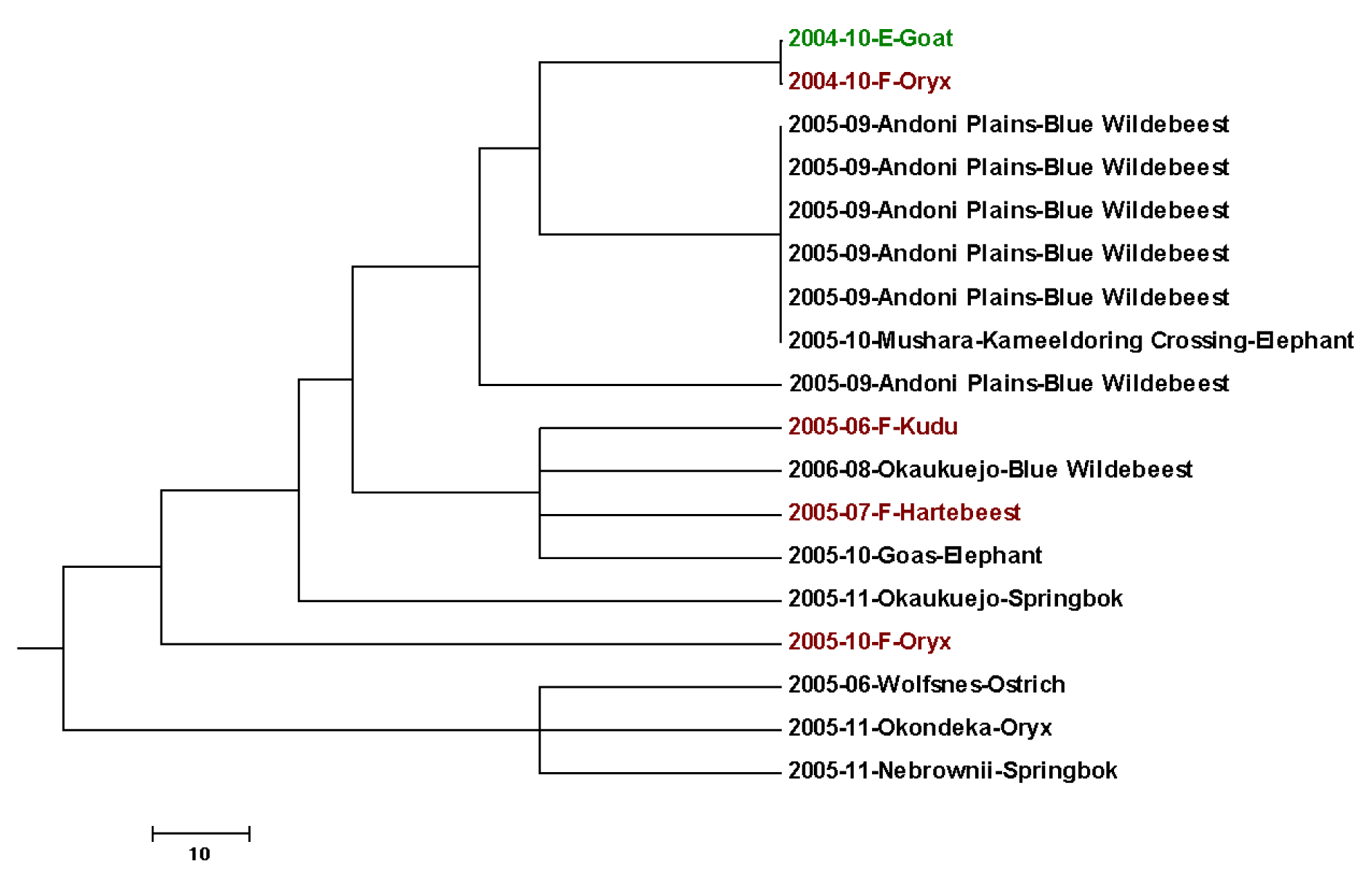

Supplement: Figure S5 — Cluster analysis of SNR data from all isolates of GT22. To protect the privacy and security of farm owners, the identities of farms are designated simply by upper case letters. (TIF) [file pntd.0001534.s005.tif]

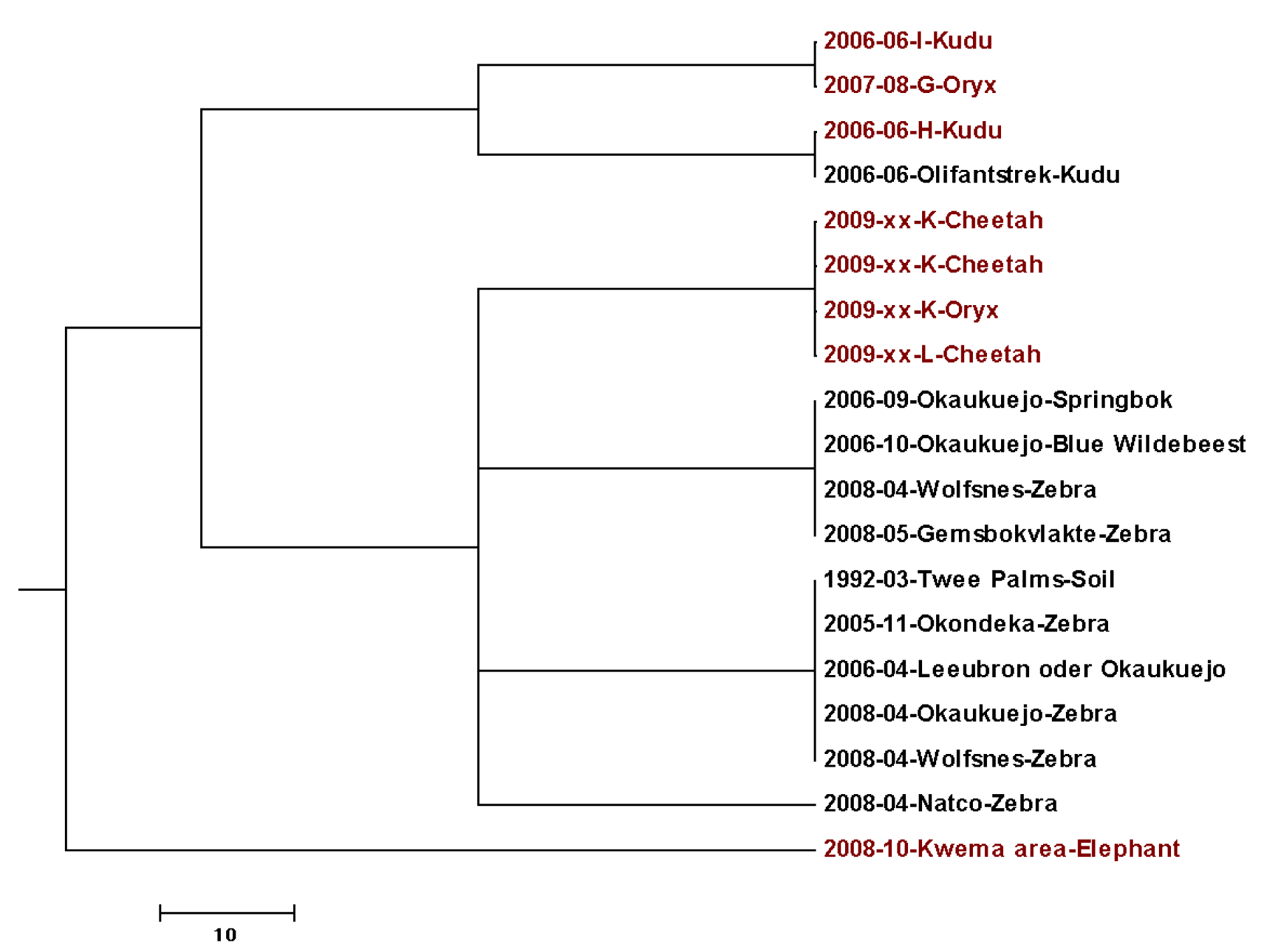

Supplement: Figure S6 — Cluster analysis of SNR data from all isolates of GT9. To protect the privacy and security of farm owners, the identities of farms are designated simply by upper case letters. (TIF) [file pntd.0001534.s006.tif]
