# Supplementary material for: Distribution and Molecular Evolution of Bacillus anthracis Genotypes in Namibia
Source: PLoS Negl Trop Dis. 2012 Mar 6;6(3):e1534. doi: 10.1371/journal.pntd.0001534 (PMC3295808; doi:10.1371/journal.pntd.0001534)
Supplement: Table S3 — Records on ENP isolates used for outbreak analysis. (PDF) [file pntd.0001534.s009.pdf]

Table S2: Records on isolates of the ENP used for outbreak analysis

Isolate No.	MLVA-Genotype	Species	Code	Origin	Comment
**YEAR 1983**					
ASC58	6	Elephant	N8K0Q93B	Gemsbokvlakte, road to Olifantsbad	
ASC59	6	Elephant	N8K0Q93L	Rhinodrive	
ASC60	6	Elephant	N8K0Q93V	Gobaub	
ASC61	6	Burchell's zebra	N8K0Q945	Gemsbokvlakte	
ASC62	6	Burchell's zebra	N8K0Q94F	Gemsbokvlakte	
**YEAR 1987**					
ASC72	6	Burchell's zebra	N8K0Q971	not known	
ASC73	6	Burchell's zebra	N8K0Q97H	not known	
ASC74	17	vulture feces	N8K0Q97N	near zebra ASC72	
ASC75	4	Blue wildebeest	N8K0Q97Y	not known	
ASC76	4	Springbok	N8K0Q98B	not known	
ASC77	**5**	Blue wildebeest	N8K0Q98L	not known	
**YEAR 1988**					
AF73	14	Elephant	881013 RV	1 km NW Pionier dam	continuing outbreak
AF79	14	Elephant	881106 RV	Duikerdrink	
AF80	14	Elephant	881203 LH	Fisher's Pan	**possible Elephant movement, but not yet supported by tracking data**
AF77	29	Elephant	881026 RV	1 km E turnoff Starks Mt.	
AF69	6	Hartmann's Zebra	880325 DJG	Starks Mt.	probably continuing or recurrent outbreak(s)
AF70	6	Hartmann's Zebra	880521 AdT	Ratelfdraf turnoff	
AF68	6	Blue wildebeest	880530 ML	14 km N Okondeka	
AF71	6	Elephant	880911 RV	400 m S Rateldraf turnoff	

AF78	6	Elephant	881018 RV	Dolmietpunt gravel pit	
AF74	6	Elephant	881020 DJG	Dolomietpunt	
AF75	6	Elephant	881026 RV	1 km E turnoff Starks Mt	
AF76	6	Elephant	881029 WH	Chudop	**possible Elephant movement, but not yet supported by tracking data**
**YEAR 1989**					
AF81	14	Elephant	890918 ML	Okawao	probably continuing outbreak
AF83	14	Elephant	891208 SSS	1 km S Tobieroen	
AF86	14	Elephant	891204	Tobieroen	
AF88	14	Elephant	891208 FJ	Tobieroen	
AF89	14	Elephant	891208 FJ	Nerens	
AF91	14	Elephant	891208 SSS	1 km S of Nerens	
AF92	14	Elephant	891208 SSS	0.5 km W of Nerens	
AF94	14	Elephant	891114 MdP	no record	
AF87	15	Elephant	891204	Tobieroen	
AF93	6	Elephant	891114 MdP	no record	
**YEAR 1991**					
AF 96	4	Elephant	91.7.11.RD	14 km NW Halali	
AF 99	4	Burchell's zebra	91.9.1.WCG	Okondeka, 2 km W	
AF 101	4	Blue Wildebeest	91.12.30.JLR	3 km E of Namutoni	separate outbreak
AF 95	6	Burchell's zebra	91.4.25 WCG	N of Wolfsnes	
AF 98	6	Blue Wildebeest	91.9.1.WCG	5 km W of Kameeldoring	separate outbreak
**YEAR 1992**					
AF105	6	Burchell's zebra	920216-01 JLR	Namutoni, Blankshelm	probably the same outbreak
AF 106	6	Blue wildebeest	920219-01 JLR	Namutoni	
AF 107	6	Blue wildebeest	920325-01 JLR	Namutoni airstrip	
AF 116	6	Elephant	920419-01 JLR	4 km N of Mushara	possible Elephant movement
AF 121	6	Blue wildebeest	920929-01 JLR	Chudop	continuing or recurrent outbreak
AF 124	6	Burchell's zebra	92106-01 JLR	1 km S of Namutoni	

AF 128	6	Elephant	921216-01 WV	Pan point	possible Elephant movement
AF 108	23	Elephant	920313-01 MA	no record	
AF 111	23	Elephant	920411-01 WDP	Gobaub	same outbreak possibly disseminated
AF 112	23	Elephant	920415-01 RD	13 km N of Halali	
AF 113	23	Elephant	920422-02 BF	0.5 km S of Gemsbokvlakte	
AF 28	9	Blue wildebees	920314-01 JLR	2 km W of Twee Palms	separate outbreak
AF 104	4	Elephant	920215-01 JLR	Namutoni sewage farm	separate outbreak
AF 109	4	Blue wildebeest	920314-01 JLR	2 km W of Twee Palms	
AF123	3	Blue wildebeest	921012-01 JLR	N edge Fishers pan	separate outbreak
AF 119	7	Blue wildebeest	92817-01 JLR	2 km NE of Namutoni	separate outbreak
AF 103	17	Blue wildebeest	92219-01 JLR	Namutoni	separate outbreak
**YEAR 1994**					
AF 133	6	Springbok	94.10.05.MA	Koinachas	
AF 134	23	Elephant	94.11.24.GM	Rhino drive	
**YEAR 1995**					
AF 136	6	Burchell's zebra	95.07.19.PML	3.5 km along W Ombika detour	
AF 137	6	Blue Wildebeest	95.09.18.FS	1 km E of Gobaub turnoff. Next to road.	
AF 130	6	Burchell's zebra	95.11.2.VM	W of Halali on loop road 15m N of road	
AF 141	14	Burchell's zebra	95.09.22.FS	5 km S of Halali turnoff	
**YEAR 2002**					
AF 3	10	Lion	SJ 2815 OVC 2815/09/02	**Sonderkop**	
AF 4	19	Antelope	SJ 2815 OVC 2844/09/02	**Okaukuejo, 12 km N**	
**YEAR 2005**					
AF48	22	Ostrich	050630 WV	Wolfsnes 2.5 km SW	
AF 56	22	Blue wildebees	050919 BK	Andoni Plains	

AF 58	22	Blue wildebeest	050919 BK	Andoni Plains	probably the same outbreak
AF 59	22	Blue wildebeest	050919 BK	Andoni Plains	
AF 60	22	Blue wildebeest	050919 BK	Andoni Plains	
AF 61	22	Blue wildebeest	050919 BK	Andoni Plains	
AF 62	22	Blue wildebeest	050919 BK	Andoni Plains	
AF 32	22	Elephant	051001 WV	Mushara/Kameeldoring crossing	
AF 31	22	Elephant	051005-01 IU	Goas	**possible Elephant movement, but not yet supported by tracking data**
AF33	22	Springbok	051101 WV	Okaukuejo Airfield 5.5 km N	continuing or recurrent outbreak
AF44	22	Gemsbock	051105-02 WK	Okondeka N	
AF36	22	Springbok	51106	Nebrownii	
AF17	32	Bovine	9999 WVC 0517.02.05	Springbokvlakte	separate outbreak
AF52	6	Springbok	051206 WV	Okondeka, 0.8 km S	separate outbreak
AF154	9	Burchell’s zebra	051122-01 BK	Okondeka 2.64km W	separate outbreak
AF43	18	Gemsbok	051013-01	Okaukuejo 12 km N	probably the same outbreak
AF34	18	Springbok	051104 WK	Okondeka N	
AF 65	18	Burchell’s zebra	051104 WT	Okondeka 2 km W	
AF 64	18	Burchell’s zebra	051104 WV	Leeubron	
AF30	19	Elephant	050930-01 BK	Aus 2.5 km NW towards Odongab	separate outbreak
AF46	21	Burchell’s zebra	051107 WV	Okaukuejo Airfield 8. km N	separate outbreak
**YEAR 2006**					
AF 173	6	Burchell’s zebra	060220 WT	Leeubron-Adamax Two-Track-Road	probably the same outbreak
AF 153	6	Burchell’s zebra	060220 WT-01	Gravel pit W of Leeubron	
AF 198	6	Burchell’s zebra	060308 WT	Adamax gravel pit 1.93 km S	
AF 164	6	Burchell’s zebra	060314-03 BK	Natco 5.18 km NE on two-track road	
AF 166	6	Springbok	060315-01 BK	Leeubron 5.29 km N on two-track road	
AF 160	6	Burchell’s zebra	060318-02 WV	Okondeka 4.55 km W	
AF 163	6	Burchell’s zebra	060318-01 WV	Okondeka 4.65 km W	

AF 165	6	Springbok	060320 WT	Leeubron, 5.22 km NW on 2-spoor track	outbreak, continuing
AF 158	6	Burchell’s zebra	060328-01 or 03 WT	Leeubron 3.96 km NW of Adamax Gravel pit, 1,89 km W	
AF 174	6	Burchell’s zebra	060328-02 WT	Leeubron, 3.74 km NW	
AF 169	6	Springbok	060330 WT	Adamax 4.67km SE	
AF 170	6	Burchell’s zebra	060331-01 WT	Adamax gravel pit 2.17 km NW	
AF 159	6	Burchell’s zebra	060406-01 or 03 WT	Leeubron 2.55 km SE of Airstrip 0,74 km W	
AF 162	6	Burchell’s zebra	060412-01 WV	Namutoni 6.7 km W	probably separate outbreak
AF 157	6	Burchell’s zebra	060413-01 JK	Natco - Adamax	continuing from before
AF 156	6	Burchell’s zebra	060512-02 or -03 WV	Okondeka 8.52 km W of Adamax gravel pit, 1,25 km S	
AF 155	6	Springbok	060603 WT	Dolomietpunt	probably separate outbreak
AF 188	6	Burchell’s zebra	061011 WK-01	Gemsbokvlakte 2 km NW	recurrent outbreak or continuing from before
AF 189	6	Springbok	061016 GS-01	Okaukuejo airstrip, 2.5 km N	
AF 194	6	Burchell’s zebra	061017 WT	Okaukuejo Airstrip 2.71 km W	
AF 161	9	Burchell’s zebra	060309 WT	Adamax gravel pit, 0.5 km SE	probably the same outbreak
AF172	9	Burchell’s zebra	060406-01 or 03 WT	Leeubron 2.55 km SE or Airstrip 0,74 km W	
AF 191	9	Springbok	060929-01 WK	Okaukuejo 3KM E	continuing or recurrent
AF 197	9	Blue wildebees	061008 WV	Okaukuejo	
AF 168	14	Burchell’s zebra	060331-02 WT	Adamax gravel pit 3.12 km NW	separate outbreak
AF 52	22	Blue wildebees	060313 WV	Okaukuejo 8 km towards Leeubron	separate outbreak
AF 171	4	Burchell’s zebra	060330 WT	Leeubron 5.34 km W	separate outbreak
AF 192	4	Burchell’s zebra	061130-01 SK	Pan road	
			**YEAR 2007**		
AF 185	6	Springbok	070220 WT	Okaukuejo E	probably the same
AF 186	6	Elephant	070503 WT	Charls Marais dam	
AF 181	6	Burchell’s zebra	070516 SK	Leeubron detour	
AF 177	6	Burchell’s zebra	070606 WV	Eindpaal 400 m N of trough	
AF 176	6	Blue wildebees	070607 WV	Leeubron 1.72 km S	

AF 193	6	Blue wildebeest	070819 WV	Okondeka 700 m N	outbreak, continuing
AF 196	6	Black rhinoceros	70820	Pan Point	
AF 150	6	Springbok	070919-01 WV	Okaukuejo 9.4 km towards Leeubron	
AF 183	6	Burchell's zebra	070925 WV	Adamax gravel pit 1.8 km SE	
AF 205	6	Burchell's zebra	071010-01 WV	Duineveld 4.8 km E	probably separate outbreak
AF 206	2	Burchell's zebra	071113-01 WV	Okaukuejo air field 500m S	
**YEAR 2008**					
AF 199	6	Springbok	080310-01 OS	Leeubron N	probably the same outbreak, continuing
AF 210	6	Burchell's zebra	080328-01 HG	Leeubron/Adamax two-track	
AF 211	6	Burchell's zebra	080330-01 OS	Natco/Adamax road	
AF 212	6	Burchell's zebra	080403-01 LP	Natco intersection	
AF 217	6	Burchell's zebra	080415-01 OS	Leeubos N	
AF 218	6	Burchell's zebra	080415-02 OS	Leeubron detour 50 m W	
AF 219	6	Burchell's zebra	080417-01 NB	Gaseb 1 km S towards Ombika	
AF 200	6	Springbok	080425-01 WV	Leeubron S	
AF 202	6	Blue wildebeest	080429-01 WV	Air Field Namutoni 6.21 km W	separate outbreak
AF 203	6	Blue wildebeest	080507-01 MK	Okaukuejo 3 km S	continuing from before
AF 201	6	Springbok	080508-01MK	Gemsbokvlakte	
AF 251	6	Elephant	080624-01TD	Nao-Obes, 1 km N, S of Halali	possible Elephant movement
AF 223	6	Springbok	080707-01 GS	Leeubos area, rd. between Leeubron & Okondeka	recurrent or continuing from before
AF 247	6	Elephant	080924-01 GS	Aroe NE	recurrent or continuing from before
AF 249	6	Elephant	081015 MK	Okaukuejo 500 m S	recurrent or continuing
AF 230	6	Springbok	081018-01 GS	Okaukuejo 1 km E	
AF 248	6	Elephant	081021-01 GS	Outside ENP at spring at Okashana centre	possible Elephant movement out of ENP
AF 222	6	Springbok	81023	King Nehale Gate along fence outside ENP	continuing from before
AF 225	6	Springbok	081106-01 NB	Nebrowni 0.8 km W	continuing from before

AF 235	6	Burchell's zebra	081106 GS	Halali, few km W	probably separate outbreak
AF 231	6	Blue wildebeest	081117 WV	Twee Palms	recurrent or continuing from before
AF 253	6	Springbok	081125-01 PA	Gemsbokvlakte E on plains	continuing from before
AF 233	6	Burchell's zebra	081128 MK	Nebrowni 1 km E	
AF 226	6	Burchell's zebra	081210 HS	Natco area	
AF 204	6	Vulture	E 094 WBV	ENP unknown	
AF 213	9	Burchell's zebra	080405-01 PK	Wolfsnes 2 km SW	probably the same outbreak
AF 214	9	Burchell's zebra	080407-01 MK	Natco/Sprokieswoud junction S	
AF 215	9	Burchell's zebra	080410-01 PK	Wolfsnes 5 km SE	
AF 216	9	Burchell's zebra	080411-01 WT	Okaukuejo air field 6.5 km N	
AF 220	9	Burchell's zebra	080508-01 OS	Gemsbokvlakte W	
AF 250	9	Elephant	080804-01 GS	Eindpaal	recurrent or continuing
AF 228	9	Blue wildebeest	080910 MK	Okakukuejo airstrip N	
AF 221	9	Elephant	081017-01 GS	Kwema area outside ENP, 12 km E of King Nehale Gate	possible Elephant movement out of ENP
AF 237	9	Springbok	081114 WV	Okaukuejo W towards shooting range	recurrent or continuing from before
AF 227	9	Burchell's zebra	081119-01 DS	Okaukuejo 3 km towards Gemsbokvlakte	
AF 232	9	Burchell's zebra	081210 HS	Natco areaA	
AF 207	4	Burchell's zebra	080305-01 OS	Leeubron area	Probably the same outbreak
AF 208	4	Burchell's zebra	080324-01 OS	Leeubron N	
AF 209	4	Burchell's zebra	080327-01 HG	Leeubron 1.39 km SW	
AF 229	4	Springbok	081018 MK	Okaukuejo-Leeubron	recurrent or continuing
AF 224	4	Gemsbok	081201 WV	Okondeka	
**YEAR 2009**					
AF 234	6	Burchell's zebra	090103-02 BK	Okaukuejo 1 km N 400 m E of road	
AF 244	6	Springbok	090120 WV	3.94 km S of Leeubron to 200m E of road	
AF 238	6	Springbok	090212 WV+RZ	3.47 km NW of Adamax on old Okahakane track	
AF 243	6	Burchell's zebra	090212 WV+RZ	2.5 km NW of Adamax on a 2 track rd ot Okahakane	
AF 257	6	Burchell's zebra	090225 RZ	Wolfsnes	

AF 258	6	Burchell’s zebra	090301-01 ZH	10km S of Leeubron on main road from Okaukuejo	
AF 259	6	Springbok	090302-01 ZH	Leeubron	
AF 260	6	Burchell’s zebra	090303-02 MK	NW of Okaukuejo, road to Leeubron	
AF 261	6	Springbok	090307-01 SB	Gravel Pit W of road to Leeubron	
AF 262	6	Springbok	090308-01 ZH	W of Leeubron	
AF 263	6	Burchell’s zebra	090311-01 MK	890 m NW of Leeubron, 70 m S of road	
AF 264	6	Burchell’s zebra	090312-01 MK	990 m SW of SA junction, 120 m S of road	
AF 310	6	Burchell’s zebra	090318-01 SB	SW of Leeubron	
AF 265	6	Burchell’s zebra	090324-01 ZH	W of Leeubron	
AF 311	6	Springbok	090326-01 MK	5.14 km NW of Okaukuejo	
AF 267	6	Burchell’s zebra	090401-01 MK	E of Sprokieswoud	
AF 270	6	Burchell’s zebra	090406-01 MK	~5 km NW of Okaukuejo; ~10.5 km SE of Leeubron	
AF 272	6	Burchell’s zebra	090407-01 ZH	Sprokieswoud-Adamax junction	
AF 274	6	Burchell’s zebra	090408-01 ZH	SA junction	
AF 278	6	Burchell’s zebra	090410-01 MK	~200 m N of E-W runway Okaukuejo airfield	
AF 279	6	Burchell’s zebra	090410-02 MK	Airfield	
AF 280	6	Burchell’s zebra	090411-01 SB	NW of SA (Sprokieswoud-Adamax) junction	probably the same outbreak, continuing
AF 288	6	Burchell’s zebra	090417-01 MK	Betsy's corner, road to Leeubron	
AF 289	6	Burchell’s zebra	090419 ZH	E of Leeubron	
AF 294	6	Burchell’s zebra	090423-01 MK	10 km NW of Okaukuejo on road to Leeubron	
AF 292	6	Springbok	090424-01 MK	4 km N of Okaukuejo	
AF 296	6	Springbok	090427-01 MK	3 km N of SA junction	
AF 300	6	Burchell’s zebra	090514-01 ZH	eastern bend in W drive	
AF 303	6	Burchell’s zebra	090526-01 ZH	~8 km S of Leeubron by trees in bend	
AF 305	6	Burchell’s zebra	090606-01 MT	~5 km NW of Okaukuejo	
AF 308	6	Burchell’s zebra	090623-01 SB	W of SA junction	
AF 336	6	Springbok	090703-01 SB	2k W of Leeubron	
AF 320	6	Burchell’s zebra	090707-01 SB	~300m S of gravel pit on way to Leeubron	

AF 334	6	Elephant	090716-01 MK	2.5km W of Aus	
AF 327	6	Burchell’s zebra	090825-01 ZH	~2 km NE of Okaukuejo, Rd to Gemsbokvlakte	
AF 323	6	Burchell’s zebra	090914-01 PK	just W of Gemsbokvlate, near junction N to main rd	
AF 322	6	Burchell’s zebra	090921-01 MK	~5 km SE of Leeubron	
AF 321	6	Burchell’s zebra	090929-01 MK	2.1 km S of Leeubron, SW of detour Rd	
AF 330	6	Burchell’s zebra	090929-01 WV	hill to NW of airfields	
AF 329	6	Burchell’s zebra	091001-01 HG	1.79 NNE of Okaukuejo	
AF 335	6	Burchell’s zebra	091115-01 MK	just S of Okaukuejo waterhole	
AF 318	6	Burchell’s zebra	091118-01 MK	4.6 km E of Sprokieswoud	
AF 319	6	Burchell’s zebra	091119-01 ZH	~ 2 km W of Leeubron	
AF 316	6	Burchell’s zebra	091120-01 ZH	turn-off to gravelpit S of Leeubron	
AF 353	6	Springbok	091201-01 MK	750 m NEN of Okaukuejo, rd to gravelpit by big Acacia	
AF 354	6	Burchell’s zebra	091203-01 MK	~3 km E of Kapupuhedi, 600m S of Road	
AF 252	6	Vulture	wingtag E025	Twin Trees, N of Okaukuejo	
AF 236	4	Burchell’s zebra	090103-01 BK	Okaukuejo airfield SE end of SE/NW strip	
AF 245	4	Springbok	090121-01 MK	~100 m W of junction two track to Grunewald	
AF 266	4	Burchell’s zebra	090327-01-MK	NW of Natco	
AF 287	4	Burchell’s zebra	090404-01TK	Fisher's pan, Namutoni	probably separate outbreak
AF 268	4	Burchell’s zebra	090405-01 MK	Grootvlakte, S Woud, ~150 m SW of horse camp	
AF 269	4	Burchell’s zebra	090405-02 MK	Gravel pit N of Grunewald	
AF 273	4	Burchell’s zebra	090407-02 ZH	Sprokieswoud-Adamax junction	
AF 275	4	Burchell’s zebra	090408-02 ZH	gravel pit by Adamax	
AF 276	4	Burchell’s zebra	090409-02 SB	West of Leeubron	
AF 277	4	Burchell’s zebra	090409-03 ZH	Okondeka-Adamax gravel pit	
AF 281	4	Burchell’s zebra	090411-01 ZH	SW of SA junction	
AF 282	4	Burchell’s zebra	090411-02 MK	SE of Grootvlatke horse camp	probably the

AF 283	4	Burchell’s zebra	090414-01 MK	9 km N of Okaukuejo	same outbreak, continuing
AF 284	4	Burchell’s zebra	090415-01 ZH	4 km NW of Okaukuejo	
AF 285	4	Burchell’s zebra	090416-01 ZH	Adamax-triangle, western 2 tracks	
AF 290	4	Burchell’s zebra	090421-01 SB	N side of western 2 track	
AF 295	4	Springbok	090424-01 SB	W of SA junction, N side of road	
AF 301	4	Springbok	090522-01 MK	2.39 km NE of Okaukuejo, S of west - east runway airfield	
AF 307	4	Burchell’s zebra	090616-01 JK	Nomab parking area in a road	
AF 328	4	Elephant	090716-01 MK	Gemsbokvlakte waterhole, 5 m E of trough	
AF 241	9	Springbok	090119-01 MK	Main road to Leeubron, near springbok stuck in a tree in October 2008	probably the same outbreak, continuing
AF 246	9	Springbok	090121-02 MK	NW of Okaukuejo	
AF 242	9	Blue wildebeest	090123-01 ZH	4.3 km W of Leeubron, ~70 m S of road	
AF 291	9	Burchell’s zebra	090422-01 MK	700 m S of Gemsbokvlakte at 10 m from road	recurrent or continuing outbreak
AF 297	9	Burchell’s zebra	090504-01 MK	~370 NW of Ombika waterhole (western detour)	
AF 299	9	Blue wildebeest	090507-01 MK	2.51 km NW of Okaukuejo	
AF 326	9	Burchell’s zebra	090716-01 MK	~3 km NW of Okaukuejo	
AF 333	9	Burchell’s zebra	090901-01 ZH	~1-2 km S of Okaukuejo, rd to Ombika	
AF 332	9	Burchell’s zebra	090914-01 MK	4.96 km NW of Okaukuejo	
AF 317	9	Springbok	091119-01 WV	1.4 km N of Okaukuejo	recurrent or continuing from before
AF 239	5	Burchell’s zebra	090123 WT	S of Leeubron	
AF 304	5	Springbok	090604-01 RZ	Halali	probably separate outbreak
AF 254	5	Vulture	wingtag E025	Twin Trees, N of Okaukuejo	
AF 293	8	Burchell’s zebra	090424-01 MK	3.5 km NW of Okaukuejo	continuing

AF 302	8	Springbok	090522-02 MK	2.07 NE of Okaukuejo SW of N-runway	outbreak
AF 309	25	Springbok	090626-01 ZH	W-drive north of Gemsbokvlakte ~ 15 km	separate outbreak
AF 355	16	Burchell's zebra	091213-01 BK	2km E of Okaukuejo, ~200 m S of main road	separate outbreak
AF 298	3	Burchell's zebra	090505-01 MK	~800 m W of Okondeka, road to Adamax	separate outbreak
**YEAR 2010**					
AF 357	4	Burchell's zebra	100201-02 SB	2 km north of twin trees	
AF 360	4	Burchell's zebra	100202-01 MK	3.95 km north-west of Gemsbokvlakte, road between Gemsbokvlakte & main road Halali, 450 m east of road	
AF 359	4	Burchell's zebra	100202-02 SB	~1 km south of Leeuboss	
AF 361	4	Burchell's zebra	100202-02 MK	1.12 km north of Okaukuejo waterhole, 150 m east of road to airfield	
AF 362	4	Burchell's zebra	100203-08 MK	5.23 km north-west of Okaukuejo, east of 2-track going south from main road to Leeubron	
AF 363	4	Burchell's zebra	100203-07 MK	~3 km north-west of Okaukuejo, north of sewer road	
AF 365	4	Burchell's zebra	100203-04 MK	2.5 km north-west of Okaukuejo	
AF 369	4	Burchell's zebra	100204-03 SB	north of 2-track to Grunewald about 1 km west of main road to Leeubron	
AF 371	4	Burchell's zebra	100204-02 SB	300 m east of main road to Leeubron and 6.13 km north-west of Okaukuejo	
AF 372	4	Burchell's zebra	100205-01 ZH	east of main road near sewer road	

AF 375	4	Burchell’s zebra	100208-02 SB	2.96 km north-west of Gemsbokvlakte, 100 m north of main road to Gemsbokvlakte	continuing outbreak
AF 383	4	Burchell’s zebra	100219-02 MK	1st gravel pit (100 m north-west of gravel pit) on way from airfield to Leeubron	
AF 386	4	Burchell’s zebra	100219-01 SB	10.7 km north of airfield turnoff, 55 m east of road	
AF 382	4	Burchell’s zebra	100219-01 MK	6.6 km north-west of Okaukuejo, road to Leeubron (after betsy's turn)	
AF 385	4	Burchell’s zebra	100219-02 SB	1.24 km west of Okondeka 1 m north of road	
AF 388	4	Burchell’s zebra	100222-04 SB	7.56 km south-west of Wolfnes, ~2 km east of Leeubron road & ~km west of Okondeka road	
AF 390	4	Blue wildebeest	100224-01SB	~70 m west of Okondeka road, ~300 m north of airfield turnoff	
AF 392	4	Burchell’s zebra	100224-02 WT	eastern 2-track in triangle, 100 m east of road	
AF 394	4	Burchell’s zebra	100225-01 SB	~3 km south of Gaseb on west-drive, ~150 m west of road	
AF 395	4	Burchell’s zebra	100228-01 MK	960 m north-east of Etosha Ecological Institute (EEI), ~400 m east of gravel pit	
AF 396	4	Burchell’s zebra	100301-01 MK	2.73 km south-east of Adamax, 20 m off road eastside	
AF 397	4	Burchell’s zebra	100301-01 SB	5.5 km west of Okaukuejo	
AF 398	4	Burchell’s zebra	100302-01 SB	~3 km north of Natco, ~200 m west of road	
AF 404	4	Burchell’s zebra	100317-02 SB	Okaukuejo waterhole	
AF 402	4	Burchell’s zebra	100317-01 MK	4.52 km north-west of Okaukuejo, north of gravel pit across from north end of sewage 2-track	

AF 405	4	Burchell's zebra	100320-01 SB	5.41 km north of Okaukuejo, between Leeubron & Okondeka roads	
AF 407	4	Burchell's zebra	100321-01 SB	2.9 km north-north-east of Okaukuejo	
AF 409	4	Burchell's zebra	100323-02 MK	3.2 km north-west of Okaukuejo, road to Leeubron, 285 m east of sewage 2-track	
AF 410	4	Burchell's zebra	100323-01 SB	4.26 km north-west of Wolfnes, ~740 m north-west of Wolfsnes-Leeubron road	
AF 413	4	Burchell's zebra	100323-05 SB	512 m east of road, road to Leeubron, 6.91 km north-west of Okaukuejo	
AF 411	4	Burchell's zebra	100323-03 SB	4.8 km north-west of Leeubron, 458 m east of Leeubron 2-track	
AF 415	4	Burchell's zebra	100324-01 MK	2.5 km north of Okaukuejo, 1 km east of road to airfield	
AF 418	4	Burchell's zebra	100325-02 MK	723 m south of Okondeka, 160 m west of road	
AF 420	4	Burchell's zebra	100330-01 SB	400 m south of Sprokieswoud-Adamax junction	
AF 424	4	Burchell's zebra	100402-01 SB	1.24 km north of Natco, 180 m east of road	
AF 425	4	Burchell's zebra	100406-02 SB	7.45 km east of Adamax, 122 m north of road	
AF 356	6	Burchell's zebra	100115-01 MK	2.67 km east of Leeubron, south of Leeubron-Okondeka road	
AF 358	6	Springbok	100201-02 ZH	where western 2-track meets with Adamax-Okondeka road	
AF 364	6	Burchell's zebra	100203-05 MK	~4 km north-west of Okaukuejo off of sewer road	
AF 366	6	Burchell's zebra	100203-03 MK	~400 m north-east of East-West runway	
AF 367	6	Burchell's zebra	100203-02 MK	0.75 km north of East-West runway	

AF 368	6	Burchell's zebra	000203-01 MK	3.33 km north-west of Okaukuejo, ~50 m east of East-West runway	continuing outbreak
AF 370	6	Burchell's zebra	100204-04 SB	north-east of Leeubron on road to Wolfsnes, south side of road	
AF 373	6	Burchell's zebra	100205-01 MK	3.22 km north-east of Okaukuejo, 980 south-east of East-West Runway	
AF 374	6	Springbok	100208-01 MK	600 m north of Airfield, 3.6 km north of Okaukuejo	
AF 376	6	Springbok	100209-01 MK	2.2 km north of exclosure (eastern 2-track)	
AF 377	6	Burchell's zebra	100209-01 MK	3.62 km south-east of Leeubron, 600 m west of main road	
AF 378	6	Blue wildebeest	100212-01 SB	0.76 north-west of Gemsbokvlakte	
AF 379	6	Burchell's zebra	100215-01 MK	~4 km east of Sprokieswoud-Adamax junction	
AF 380	6	Burchell's zebra	100218-01 SB	~5 km west of Adamax on old track	
AF 384	6	Burchell's zebra	100219-02 WT	5.6 km north-west of Okaukuejo, ~200 m west of road to Leeubron, south of two track to Grunewald	
AF 389	6	Burchell's zebra	100222-01 SB	880 m east of road, 2.8 km south-west of Wolfsnes	
AF 393	6	Springbok	100223-01 WT	1 km north of Wolfsnes turnoff on side of road, 0.5 m off road	
AF 391	6	Burchell's zebra	100224-01 WT	370 m west of Leeubron detour, 630 m south of Leeubron-Sprokieswoud-Adamax road	
AF 399	6	Blue wildebeest	100301-01 BK	1 km from Namutoni towards Causeway	separate outbreak
AF 400	6	Burchell's zebra	100307-01 SB	~300 m south of main road, ~ 1 km east of Gemsbokvlakte turnoff (1st one)	

AF 403	6	Burchell's zebra	100317-01 SB	5.83 km north-north-west of Okaukuejo, 1.27 km west of road to Okondeka, west of first Acacia on road north	continuing from before
AF 406	6	Burchell's zebra	100320-02 SB	5.04 km north of Okaukuejo, between Leeubron & Okondeka roads	
AF 414	6	Springbok	100323-02 SB	300 m north-west of Leeuboss	
AF 412	6	Burchell's zebra	100323-04 SB	4.45 km south-west of Leeubron, 1.21 km east of SA junction road	
AF 408	6	Burchell's zebra	100323-01 MK	3.2 km north of Okaukuejo, 420 m south of East-West Runway	
AF 417	6	Blue wildebeest	100324-01MK	4.2 km north-north-east of Okaukuejo, 469 m north-north-east of East-West runway, 796 m south-west of North-South runway (North end)	
AF 419	6	Burchell's zebra	100325-03 MK	890 m south-west of Okondeka, 608 m west of road	
AF 422	6	Burchell's zebra	100331-01 SB	2.4 km west of Leeubron, 750 m north of road	
AF 421	6	Burchell's zebra	100331-01 ZH	81 m east of Eastern-2-track (368 m north of Leeubron-Okondeka road)	
AF 423	6	Springbok	100402-01 SB	5.07 km east of Adamax, 155 m south of road	
AF 431	6	Springbok	100409-01 MK	2.1 km north of Okaukuejo road to airfield, 300 m east of road	
AF 416	8	Springbok	100324-01 MK	5 km south-west of Leeubron, 560 m west of road	separate outbreak

AF 387	9	Springbok	100220-01 MK	6.2 km north-west of Okaukuejo, ~250 m north of Grunewald two track off road to Leeubron	separate outbreak
AF 381	35	Burchell's zebra	100219-01 WT	3.2 km east-north-east of Leeubron, 520 m south of road Leeubron-Okondeka	separate outbreak
AF 401	37	Burchell's zebra	100311-02 SB	2.1 km east of Okaukuejo, ~300 m north of Gemsbokvlakte road	separate outbreak
AF 434		Burchell's zebra	100221-02 SB	Okondeka road, about 6.7 km north of airfield turnoff, 20 m west of road	
AF 436		Burchell's zebra	100221-03 SB	7.98 km from Okaukuejo, on road to Leeubron, ~30 m west of road	
AF 437		Burchell's zebra	100301-01 ZH	Sprokieswoud, north side of road across from fenced area	
AF 435		Burchell's zebra	100306-01 SB	5.2 km north-north-east Natco, 195 m west of road	
AF 438		Burchell's zebra	100311-01 SB	1.4 km east of Okaukuejo	
AF 439		Burchell's zebra	100319-01 ZH	~4 km south of Leeubron, east side of road	
AF 442		Burchell's zebra	100319-02 ZH	Okaukuejo, ~1 km north of twin trees on west side of road	
AF 426		Burchell's zebra	100408-01 SB	450 m south of Okaukuejo waterhole, 140 m south of road to water tower	
AF 427		Burchell's zebra	100408-02 SB	7.09 km south-south-west of Leeubron	
AF 428		Burchell's zebra	100408-03 SB	6.53 km south-south-west of Leeubron	
AF 429		Burchell's zebra	100408-04 SB	8.03 km north-west of Okaukuejo	

AF 432		Burchell’s zebra	100409-03 SB	~2 km west-south-west of Leeubron, ~100 m south of road to Sprokieswoud-Adamax	
AF 430		Burchell’s zebra	100409-01 ZH	open plain between Leeubron & Sprokieswoud-Adamax junction, ~2 km west of Leeubron	
AF 443		Burchell’s zebra	100411-01 MK	2 km south-east of Sprokieswoud-Adamax junction, 360 m south off road	
AF 433		Burchell’s zebra	100411-02 MK	track to Adamax gravel pit, gravel pit 3 m west of road (350 m north of gravel pit)	
AF 444		Burchell’s zebra	100412-01 ZH	800 m east of Sprokieswoukd-Adamax junction	
AF 445		Burchell’s zebra	100413-01 SB	~500 m north of road, 3.06 km west of Sprokieswoud-Adamax junction	
AF 446		Blue wildebeest	100413-01 BK	~1 km east of Sprokieswoud, ~ 300 m north of road	
AF 447		Burchell’s zebra	100413-02 SB	~100 m north-east of Grunewald gravel pit	
AF 448		Black rhinoceros	100413-01 MK	832 m north-north-east of Homob Toilet camp, in gravel pit	
AF 451		Burchell’s zebra	100414-01 SB	7 km north of Okaukuejo, ~660 m west of Okondeka road	
AF 452		Burchell’s zebra	100414-02 SB	6.21 km N of Leeubron, 245m W of Leeubron 2-track	
AF 450		Blue wildebeest	100414-01 SB	7.88 km south-east of Leeubron, 400 m south of road	
AF 449		OM	100414-01 SB	5.5 km north-west of Okaukuejo, 1.65 km east of road to Leeubron	
AF 453		Burchell’s zebra	100414-04SB	1 km north-east of Sprokieswoud-Adamax junction, ~800 m east of road to Adamax	
AF 454		Springbok	100415-02 SB	1.79 km south of Sprokieswoud-Adamax	

AF 455		Burchell's zebra	100415-01 SB	3.51 km south-west of Leeubron, 1 km south of Sprokieswoud-Adamax, 641 m west of road	
AF 456		Burchell's zebra	100415-02 SB	1.19 km south-west of Sprokieswoud-Adamax, 872 m south of road	
AF 457		Burchell's zebra	100415-03 SB	1.92 km south of Sprokieswoud-Adamax, 1.11 km west of road	
AF 459		Burchell's zebra	100416-02 SB	~100 m north of Okondeka-Adamax road, ~9 km west of Okondeka	
AF 458		Burchell's zebra	100416-01 SB	1 km west of Adamax gravel pit, ~600 m south of Okondeka-Adamax road	
AF 461		Burchell's zebra	100421-02 SB	5.02 km south-west of Natco, 1.69 km south-west of road to Sprokieswoud-Adamax	
AF 460		Burchell's zebra	100421-01 SB	4.46 km north-west of Okaukuejo	
AF 463		Burchell's zebra	100423-02 ZH	north of Okaukuejo, 2 km from twin trees	
AF 462		Burchell's zebra	100423-01 ZH	~2 km south of Wolfsnes, ~80 m north of two Acacia on the road to Okondeka	
AF 464		Blue wildebeest	100424-01 ZH	north of Okaukuejo, ~2 km from Okaukuejo and about 800 m north of sewage turnoff	
AF 465		Burchell's zebra	100427-01 ZH	890 m north-west of Okaukuejo, west of road to sewage	
AF 467		Springbok	100428-01 MK	800 m east of Sprokieswoud, 80 m north of road	
AF 466		Blue wildebeest	100428-01 MK	5.1 km west of Okondeka, ~210 m north of road to Adamax	
AF 468		Burchell's zebra	100502-01 WB	8.2 km north-west of Okaukuejo, road to Okondeka, ~20 m west of road	

AF 470		Burchell’s zebra	100503-03 MK	4.6 km south of Okaukuejo, Stark's pan, 130 m east of road	
AF 469		Burchell’s zebra	100503-01 MK	Okaukuejo-Okondeka-Leeubron junction	
AF 472		Springbok	100506-01	~4 km south of Leeubron and 100 m north of the vulture roosting trees	
AF 471		Springbok	100509-01 MK	410 m east of northern end of North-South-Runway	
AF 473		Burchell’s zebra	100510-01 MK	4.5 km west-north-west of Okaukuejo, 800 m north of two track to Grunewald	
AF 474		Burchell’s zebra	100525-01 CC	north-east of Gemsbokvlakte, 5 km from Gemsbokvlakte close to the end of transect 34	
AF 475		Burchell’s zebra	100531-01 MK	3.1 km south of Gaseb, west drive, east of road	
AF 476		Burchell’s zebra	100602-01 MK	15.4 km south-west of Gemsbokvlakte, south-east loop of west drive, 625 m west of road	
AF 477		Springbok	100603-01 MK	940 m south of Gaseb, 370 m west of West-Drive-Road	
AF 478		Burchell’s zebra	100603-01 MK	3.4 km south of Gemsbokvlakte, 550 m west of West-Drive	
AF 479		Burchell’s zebra	100607-02 MK	750 m south of Gemsbokvlakte, ~300 m east of road	
AF 480		Burchell’s zebra	100607-03 MK	1.2 km south of Gemsbokvlakte, West-Drive, ~50 m west of road in Catophractes	
AF 481		Burchell’s zebra	100607-04 MK	400 m south of Gemsbokvlakte, road to W-Drive, east of road 34 m	
AF 506		Elephant	100606-01 OA	Aus waterhole	
AF 505		Springbok	100628-01 MK	620 m north-east of Newbrownii, 200 m north of road	

AF 483		Burchell’s zebra	100614-01 MK	Diamond drive, top intersection, 265 m south of main road to Newbrownii	
AF 496		Burchell’s zebra	100617-01 MK	2.3 km north-west of Gemsbokvlakte	
AF 484		Burchell’s zebra	100618-01 SB	2 km away from Okaukuejo on road to Gemsbokvlakte	
AF 485		Burchell’s zebra	100621-01 ZH	W-Drive, east of Gaseb, ~4 km away	
AF 486		Burchell’s zebra	100622-01 WV	Halali plains west	
AF 487		Burchell’s zebra	1000625-01 SB	W-Drive ~1 km from Gemsbokvlakte, 50 m north of road	
AF 488		Burchell’s zebra	100628-01 ZH	Gemsbokvlakte 200 m from waterhole	
AF 489		Burchell’s zebra	100706-01 MK	1.7 km north-east of W-Drive gravel pit, 1 km east of W-Drive-Road	
AF 490		Burchell’s zebra	100707-01 MK	1.6 km south of Gemsbokvlakte, 700 m east of road	
AF 491		Burchell’s zebra	100707-02 MK	1.2 km south of Gemsbokvlakte, ~80 m west of road, down W-Drive	
AF 494		Burchell’s zebra	100707-03 MK	990 m north of Gemsbokvlakte, road to main road, 220 m north of road	
AF 492		Springbok	100708-01 WV	2.8 km south of Gemsbokvlakte, 180 m east of road	
AF 493		Burchell’s zebra	100714-01 SB	3.37 km south of Gonob, 50 m east of track	
AF 495		Burchell’s zebra	100719-01 MK	~2.8 km east of Gaseb turn-off, road to Gemsbokvlakte, ~400 m north of road	
AF 497		Springbok	100723-01 MK	650 m south-east of Okaukuejo, south of location, 230 m east of road	
AF 498		Elephant	100726-01 ZH	700-800 m south of Okaukuejo, west of tar road	

AF 499		Springbok	100801-01 RZ	1.1 km north-north-west of Okaukuejo,~250 m north-east of gravelpit by big Acacia	
AF 500		Springbok	100811-01 MK	two-track to Grunewald	
AF 501		LA	100828-01 MK	Okaukuejo waterhole	
AF 502		Burchell's zebra	100830-02 ZH	~4 km south-east of Gemsbokvlakte, W-Drive	
AF 503		Burchell's zebra	100906-01 ZH	~150 m north of Okaukuejo waterhole	
AF 504		Elephant	100908-01 WK	700 m along eastern detour to Ombika, southern intersection	
not yet analyzed		Burchell's zebra	100916-01 WV	on powerline corner south of location??? ~700 m	
		Burchell's zebra	100922-01 WV	open plain between Okaukuejo and airfield	
		Gemsbok	100927-01 MK	3.16 km south of Okaukuejo, 650 m east of tar road	
		Blue wildebeest	100929-01 ZH	10 km south of Leeubron, south of road	
		Springbok	100930-01 ZH	~4 km south of Leeubron, west of road	
		Elephant	100930-01 ZH	100-150 m north of Duiwelsvuur	
		Elephant	100930-02 ZH	200 m east of Duiwelsvuur waterhole	
		Black rhinoceros	101012-01 BK	Nerens	
		Burchell's zebra	101012-01 MK	840 m south-south-west of Okaukuejo, south of 2-track	
		Elephant	101012-01 MK	4.64 km south-east of M'Bari, ~15m south of road	
		Giraffe	101013-01 MK	~600 m north of Okondeka waterhole	
		Burchell's zebra	101019-01 CC	3.85 km north-east of Gemsbokvlakte, road to Nebrownii	
		Burchell's zebra	101019-01 MK	4.5 km north-north-east of Okaukuejo, east of East-West runway	
		Burchell's zebra	101019-02 MK	4.3 km north-north-east of Okaukuejo, edge of pan	

		Burchell’s zebra	101019-04 MK	just east of Gemsbokvlakte	
		Burchell’s zebra	101019-05 MK	~6 km north of Okaukuejo, road to Okondeka, 50 m west of road	
		Burchell’s zebra	101021-01 ZH	just east of Gemsbokvlakte	
		Springbok	101025-01 MK	580 m south-south-west of Gemsbokvlakte, W-drive	
		Burchell’s zebra	101112-01 ZH	4.9 km north-east?? of Okaukuejo, Leeubron road	
		Black rhinoceros	101114-01 CC	1.5 km south of Gemsbokvlakte, W-drive	

Isolate No.	MLVA-Genotype	Species	Code	Origin	Comment
**YEAR 1983**					
ASC58	6	Elephant	N8K0Q93B	Gemsbokvlakte, road to Olifantsbad	
ASC59	6	Elephant	N8K0Q93L	Rhinodrive	
ASC60	6	Elephant	N8K0Q93V	Gobaub	
ASC61	6	Burchell's zebra	N8K0Q945	Gemsbokvlakte	
ASC62	6	Burchell's zebra	N8K0Q94F	Gemsbokvlakte	
**YEAR 1987**					
ASC72	6	Burchell's zebra	N8K0Q971	not known	
ASC73	6	Burchell's zebra	N8K0Q97H	not known	
ASC74	17	vulture feces	N8K0Q97N	near zebra ASC72	
ASC75	4	Blue wildebeest	N8K0Q97Y	not known	
ASC76	4	Springbok	N8K0Q98B	not known	
ASC77	**5**	Blue wildebeest	N8K0Q98L	not known	
**YEAR 1988**					
AF73	14	Elephant	881013 RV	1 km NW Pionier dam	continuing outbreak
AF79	14	Elephant	881106 RV	Duikerdrink	
AF80	14	Elephant	881203 LH	Fisher's Pan	**possible Elephant movement, but not yet supported by tracking data**
AF77	29	Elephant	881026 RV	1 km E turnoff Starks Mt.	
AF69	6	Hartmann's Zebra	880325 DJG	Starks Mt.	probably continuing or recurrent outbreak(s)
AF70	6	Hartmann's Zebra	880521 AdT	Ratelfdraf turnoff	
AF68	6	Blue wildebeest	880530 ML	14 km N Okondeka	
AF71	6	Elephant	880911 RV	400 m S Rateldraf turnoff	

AF78	6	Elephant	881018 RV	Dolmietpunt gravel pit	outbreak(s)
AF74	6	Elephant	881020 DJG	Dolomietpunt	
AF75	6	Elephant	881026 RV	1 km E turnoff Starks Mt	
AF76	6	Elephant	881029 WH	Chudop	**possible Elephant movement, but not yet supported by tracking data**
**YEAR 1989**					
AF81	14	Elephant	890918 ML	Okawao	probably continuing outbreak
AF83	14	Elephant	891208 SSS	1 km S Tobieroen	
AF86	14	Elephant	891204	Tobieroen	
AF88	14	Elephant	891208 FJ	Tobieroen	
AF89	14	Elephant	891208 FJ	Nerens	
AF91	14	Elephant	891208 SSS	1 km S of Nerens	
AF92	14	Elephant	891208 SSS	0.5 km W of Nerens	
AF94	14	Elephant	891114 MdP	no record	
AF87	15	Elephant	891204	Tobieroen	
AF93	6	Elephant	891114 MdP	no record	
**YEAR 1991**					
AF 96	4	Elephant	91.7.11.RD	14 km NW Halali	
AF 99	4	Burchell's zebra	91.9.1.WCG	Okondeka, 2 km W	
AF 101	4	Blue Wildebeest	91.12.30.JLR	3 km E of Namutoni	separate outbreak
AF 95	6	Burchell's zebra	91.4.25 WCG	N of Wolfsnes	
AF 98	6	Blue Wildebeest	91.9.1.WCG	5 km W of Kameeldoring	separate outbreak
**YEAR 1992**					
AF105	6	Burchell's zebra	920216-01 JLR	Namutoni, Blankshelm	probably the same outbreak
AF 106	6	Blue wildebeest	920219-01 JLR	Namutoni	
AF 107	6	Blue wildebeest	920325-01 JLR	Namutoni airstrip	
AF 116	6	Elephant	920419-01 JLR	4 km N of Mushara	possible Elephant movement
AF 121	6	Blue wildebeest	920929-01 JLR	Chudop	continuing or recurrent

AF 124	6	Burchell's zebra	92106-01 JLR	1 km S of Namutoni	recurrent outbreak
AF 128	6	Elephant	921216-01 WV	Pan point	possible Elephant movement
AF 127	6	Kudu	921029	Susuwe	not ENP
AF 108	23	Elephant	920313-01 MA	no record	
AF 111	23	Elephant	920411-01 WDP	Gobaub	same outbreak possibly disseminated by Elephant movement
AF 112	23	Elephant	920415-01 RD	13 km N of Halali	
AF 113	23	Elephant	920422-02 BF	0.5 km S of Gemsbokvlakte	
AF 28	9	Blue wildebeest	920314-01 JLR	2 km W of Twee Palms	separate outbreak
AF 104	4	Elephant	920215-01 JLR	Namutoni sewage farm	separate outbreak
AF 109	4	Blue wildebeest	920314-01 JLR	2 km W of Twee Palms	
AF123	3	Blue wildebeest	921012-01 JLR	N edge Fishers pan	separate outbreak
AF 119	7	Blue wildebeest	92817-01 JLR	2 km NE of Namutoni	separate outbreak
AF 103	17	Blue wildebeest	92219-01 JLR	Namutoni	separate outbreak
**YEAR 1994**					
AF 133	6	Springbok	94.10.05.MA	Koinachas	
AF 134	23	Elephant	94.11.24.GM	Rhino drive	
**YEAR 1995**					
AF 136	6	Burchell's zebra	95.07.19.PM L	3.5 km along W Ombika detour	
AF 137	6	Blue Wildebeest	95.09.18.FS	1 km E of Gobaub turnoff. Next to road.	
AF 130	6	Burchell's zebra	95.11.2.VM	W of Halali on loop road 15m N of road	
AF 141	14	Burchell's zebra	95.09.22.FS	5 km S of Halali turnoff	
**YEAR 1998**					

AF 1	33	Bov. fed to Cheetah and Leopard	SW 0037 WWVC 1919/11/98	Okambara	not ENP
**YEAR 2000**					
AF 144	6	Burchell's zebra	Site 1 17.11.2000	Okondeka (13 yr old zebra site)	soil sample
**YEAR 2002**					
AF 2	30	Caprine	SH 938/02 BV 1946/07/02	Winkelshutten / Okahandja	not ENP
AF 3	10	Lion	SJ 2815 OVC 2815/09/02	**Sonderkop**	
AF 4	19	Antelope	SJ 2815 OVC 2844/09/02	**Okaukuejo, 12 km N**	
**YEAR 2003**					
AF 5	1	Bovine	SH 51-3 BV 2411/08/03	Okamaja/Okahandja	not ENP
AF 14	30	Caprine	SN 01/10 BM Mariental 3163/10/03	Hatzium/Mariental	not ENP
AF 15	30	Caprine	SN 03/08/2AHG 2455/08/03	Awadoab/Mariental	not ENP
**YEAR 2004**					
AF 6	1	Bovine	SO 7391 OVC 0860/03/04	Middelplaats / Otjiwarongo	not ENP
AF 7	18	Human skin	SO 2855 GE 2855/09/04	Otjiwarongo	not ENP
AF 8	18	Cheetah	SW 2043 OJB 2043/04/04	Bergzicht/Windhoek	not ENP
AF 9	18	Eland	SX 3335 FM 3335/10/04	Gobabis	not ENP
AF 10	20	Oryx	SH 3134 CL 3134/09/04	Natalia/Okahandja	not ENP
AF 11	22	Caprine	SJ 7430 OVC 3360/10/04	Nimmerus/Outjo	not ENP
AF 12	22	Oryx	SO 7429 OVC 3337/10/04	Sangaree/Outjo	not ENP
AF 13	28	Bovine	EC 001/04 JA 0059/01/04	Marasburg/East Caprivi	not ENP

AF 16	30	Caprine	SW 15335 WVC 3261/10/04	Boomplaas / Keetmanshop	not ENP
**YEAR 2005**					
AF48	22	Ostrich	050630 WV	Wolfsnes 2.5 km SW	
AF 56	22	Blue wildebees	050919 BK	Andoni Plains	probably the same outbreak
AF 58	22	Blue wildebees	050919 BK	Andoni Plains	
AF 59	22	Blue wildebees	050919 BK	Andoni Plains	
AF 60	22	Blue wildebees	050919 BK	Andoni Plains	
AF 61	22	Blue wildebees	050919 BK	Andoni Plains	
AF 62	22	Blue wildebees	050919 BK	Andoni Plains	
AF 32	22	Elephant	051001 WV	Mushara/Kameel doring crossing	
AF 31	22	Elephant	051005-01 IU	Goas	**possible Elephant movement, but not yet supported by tracking data**
AF33	22	Springbok	051101 WV	Okaukuejo Airfield 5.5 km N	continuing or recurrent outbreak
AF44	22	Gemsbock	051105-02 WK	Okondeka N	
AF36	22	Springbok	51106	Nebrownii	
AF 19	22	Kudu	7487 1906.06.05	Sangaree	not ENP
AF 20	22	Hartebeest	7498 OVC 2282.07.05	Sangaree	not ENP
AF25	22	Gemsbok	7517 OVC 3914.10.05	Sangaree	not ENP
AF51	22	Gemsbok	N (EPACHA 2-7) from 2005	Epacha farm south of Etosha border	not ENP
AF26	26	Bovine	5193 LS 51.01.12.05	Okeekolongo	not ENP
AF18	30	Caprine	11.05 YBK 0949.04.05	Ombirusu	not ENP
AF17	32	Bovine	9999 WVC 0517.02.05	Springbokvlakte	separate outbreak
AF52	6	Springbok	051206 WV	Okondeka, 0.8 km S	separate outbreak
AF154	9	Burchell's zebr	051122-01 BK	Okondeka 2.64km W	separate outbreak

AF182	13	Kudu	51219	Windport Farm 429	not ENP
AF43	18	Gemsbok	051013-01	Okaukuejo 12 km N	probably the same outbreak
AF34	18	Springbok	051104 WK	Okondeka N	
AF 65	18	Burchell's zebr	051104 WT	Okondeka 2 km W	
AF 64	18	Burchell's zebr	051104 WV	Leeubron	
AF 21	18	Gemsbok	2809 UT 2995.08.05	Bergzicht	not ENP
AF 22	18	Gemsbok	3169 CL 3169.08.05	Neu-Otjisororindi	not ENP
AF 24	18	Hartebeest	3896 CL 3896.10.05	Neu-Otjisororindi	not ENP
AF 23	18	Equine	519 WVC 3323.09.05	Nuwe Orde	not ENP
AF30	19	Elephant	050930-01 BK	Aus 2.5 km NW towards Odongab	separate outbreak
AF46	21	Burchell's zebr	051107 WV	Okaukuejo Airfield 8. km N	separate outbreak
**YEAR 2006**					
AF 173	6	Burchell's zebr	060220 WT	Leeubron-Adamax Two-Track-Road	probably the same outbreak, continuing
AF 153	6	Burchell's zebr	060220 WT-01	Gravel pit W of Leeubron	
AF 198	6	Burchell's zebr	060308 WT	Adamax gravel pit 1.93 km S	
AF 164	6	Burchell's zebr	060314-03 BK	Natco 5.18 km NE on two-track road	
AF 166	6	Springbok	060315-01 BK	Leeubron 5.29 km N on two-track road	
AF 160	6	Burchell's zebr	060318-02 WV	Okondeka 4.55 km W	
AF 163	6	Burchell's zebr	060318-01 WV	Okondeka 4.65 km W	
AF 165	6	Springbok	060320 WT	Leeubron, 5.22 km NW on 2-spoor track	
AF 158	6	Burchell's zebr	060328-01 or 03 WT	Leeubron 3.96 km NW of Adamax Gravel pit, 1,89 km W	
AF 174	6	Burchell's zebr	060328-02 WT	Leeubron, 3.74 km NW	

AF 169	6	Springbok	060330 WT	Adamax 4.67km SE	
AF 170	6	Burchell’s zebr	060331-01 WT	Adamax gravel pit 2.17 km NW	
AF 159	6	Burchell’s zebr	060406-01 or 03 WT	Leeubron 2.55 km SE of Airstrip 0,74 km W	
AF 162	6	Burchell’s zebr	060412-01 WV	Namutoni 6.7 km W	probably separate outbreak
AF 157	6	Burchell’s zebr	060413-01 JK	Natco - Adamax	continuing from before
AF 156	6	Burchell’s zebr	060512-02 or -03 WV	Okondeka 8.52 km W of Adamax gravel pit, 1,25 km S	
AF 155	6	Springbok	060603 WT	Dolomietpunt	probably separate outbreak
AF 188	6	Burchell’s zebr	061011 WK-01	Gemsbokvlakte 2 km NW	recurrent outbreak or continuing from before
AF 189	6	Springbok	061016 GS-01	Okaukuejo airstrip, 2.5 km N	
AF 194	6	Burchell’s zebr	061017 WT	Okaukuejo Airstrip 2.71 km W	
AF 161	9	Burchell’s zebr	060309 WT	Adamax gravel pit, 0.5 km SE	probably the same outbreak
AF172	9	Burchell’s zebr	060406-01 or 03 WT	Leeubron 2.55 km SE or Airstrip 0,74 km W	
AF 190	9	Kudu	060602 WK	mid dam farm	not ENP, outbreaks related to ENP
AF 195	9	Kudu	060614 WV	Epacha farm	
AF 184	9	Kudu	060618 SG TSK.	Olifantstrek area	
AF 191	9	Springbok	060929-01 WK	Okaukuejo 3KM E	continuing or recurrent outbreak
AF 197	9	Blue wildebees	061008 WV	Okaukuejo	
AF 168	14	Burchell’s zebr	060331-02 WT	Adamax gravel pit 3.12 km NW	separate outbreak
AF 52	22	Blue wildebees	060313 WV	Okaukuejo 8 km towards Leeubron	separate outbreak
AF 27	26	Bovine	5195 LS 0001/01/06	**Uutsathima**	not ENP

AF 171	4	Burchell’s zebr	060330 WT	Leeubron 5.34 km W	separate outbreak
AF 192	4	Burchell’s zebr	061130-01 SK	Pan road	
**YEAR 2007**					
AF 185	6	Springbok	070220 WT	Okaukuejo E	probably the same outbreak, continuing
AF 186	6	Elephant	070503 WT	Charls Marais dam	
AF 181	6	Burchell’s zebr	070516 SK	Leeubron detour	
AF 177	6	Burchell’s zebr	070606 WV	Eindpaal 400 m N of trough	
AF 176	6	Blue wildebees	070607 WV	Leeubron 1.72 km S	
AF 193	6	Blue wildebees	070819 WV	Okondeka 700 m N	
AF 196	6	lack rhinocero	70820	Pan Point	
AF 150	6	Springbok	070919-01 WV	Okaukuejo 9.4 km towards Leeubron	
AF 183	6	Burchell’s zebr	070925 WV	Adamax gravel pit 1.8 km SE	
AF 205	6	Burchell’s zebr	071010-01 WV	Duineveld 4.8 km E	probably separate outbreak
AF 206	2	Burchell’s zebr	071113-01 WV	Okaukuejo air field 500m S	
AF 175	2	Gemsbok	070803-01	Windport Farm	not ENP
AF 148	24	Ovine	SH 031/7 BV	Okahandja	not ENP
AF 151	9	Gemsbok	OG070802-01 WV	Windport Farm	not ENP
AF 187	9	Gemsbok	SX070802	unknown origin (Windport Farm?)	
AF 147	3	Gemsbok	SX2675CVL 07/07	Gobabis	not ENP
**YEAR 2008**					
AF 199	6	Springbok	080310-01 OS	Leeubron N	probably the same outbreak, continuing
AF 210	6	Burchell’s zebr	080328-01 HG	Leeubron/Adamax two-track	
AF 211	6	Burchell’s zebr	080330-01 OS	Natco/Adamax road	
AF 212	6	Burchell’s zebr	080403-01 LP	Natco intersection	
AF 217	6	Burchell’s zebr	080415-01 OS	Leeubos N	

AF 218	6	Burchell’s zebr	080415-02 OS	Leeubron detour 50 m W	
AF 219	6	Burchell’s zebr	080417-01 NB	Gaseb 1 km S towards Ombika	
AF 200	6	Springbok	080425-01 WV	Leeubron S	
AF 202	6	Blue wildebees	080429-01 WV	Air Field Namutoni 6.21 km W	separate outbreak
AF 203	6	Blue wildebees	080507-01 MK	Okaukuejo 3 km S	continuing from before
AF 201	6	Springbok	080508-01MK	Gemsbokvlakte	
AF 251	6	Elephant	080624-01TD	Nao-Obes, 1 km N, S of Halali	possible Elephant movement
AF 223	6	Springbok	080707-01 GS	Leeubos area, rd. between Leeubron & Okondeka	recurrent or continuing from before
AF 247	6	Elephant	080924-01 GS	Aroe NE	recurrent or continuing from before
AF 249	6	Elephant	081015 MK	Okaukuejo 500 m S	recurrent or continuing from before
AF 230	6	Springbok	081018-01 GS	Okaukuejo 1 km E	
AF 248	6	Elephant	081021-01 GS	Outside ENP at spring at Okashana centre	possible Elephant movement out of ENP
AF 222	6	Springbok	81023	King Nehale Gate along fence outside ENP	continuing from before
AF 225	6	Springbok	081106-01 NB	Nebrowni 0.8 km W	continuing from before
AF 235	6	Burchell’s zebr	081106 GS	Halali, few km W	probably separate outbreak
AF 231	6	Blue wildebees	081117 WV	Twee Palms	recurrent or continuing from before
AF 253	6	Springbok	081125-01 PA	Gemsbokvlakte E on plains	continuing from before
AF 233	6	Burchell’s zebr	081128 MK	Nebrowni 1 km E	
AF 226	6	Burchell’s zebr	081210 HS	Natco area	
AF 204	6	Vulture	E 094 WBV	ENP unknown	

AF 213	9	Burchell’s zebr	080405-01 PK	Wolfsnes 2 km SW	probably the same outbreak
AF 214	9	Burchell’s zebr	080407-01 MK	Natco/Sprokieswoud junction S	
AF 215	9	Burchell’s zebr	080410-01 PK	Wolfsnes 5 km SE	
AF 216	9	Burchell’s zebr	080411-01 WT	Okaukuejo air field 6.5 km N	
AF 220	9	Burchell’s zebr	080508-01 OS	Gemsbokvlakte W	
AF 250	9	Elephant	080804-01 GS	Eindpaal	recurrent or continuing outbreak
AF 228	9	Blue wildebees	080910 MK	Okakukuejo airstrip N	
AF 221	9	Elephant	081017-01 GS	Kwema area outside ENP, 12 km E of King Nehale Gate	possible Elephant movement out of ENP
AF 237	9	Springbok	081114 WV	Okaukuejo W towards shooting range	recurrent or continuing from before
AF 227	9	Burchell’s zebr	081119-01 DS	Okaukuejo 3 km towards Gemsbokvlakte	
AF 232	9	Burchell’s zebr	081210 HS	Natco areaA	
AF 207	4	Burchell’s zebr	080305-01 OS	Leeubron area	Probably the same outbreak
AF 208	4	Burchell’s zebr	080324-01 OS	Leeubron N	
AF 209	4	Burchell’s zebr	080327-01 HG	Leeubron 1.39 km SW	
AF 229	4	Springbok	081018 MK	Okaukuejo-Leeubron	recurrent or continuing from before
AF 224	4	Gemsbok	081201 WV	Okondeka	
**YEAR 2009**					
AF 234	6	Burchell’s zebr	090103-02 BK	Okaukuejo 1 km N 400 m E of road	
AF 244	6	Springbok	090120 WV	3.94 km S of Leeubron to 200m E of road	
AF 238	6	Springbok	090212 WV+RZ	3.47 km NW of Adamax on old Okahakane track	
AF 243	6	Burchell’s zebr	090212 WV+RZ	2.5 km NW of Adamax on a 2 track rd ot Okahakane	
AF 257	6	Burchell’s zebr	090225 RZ	Wolfsnes	

AF 258	6	Burchell’s zebr	090301-01 ZH	10km S of Leeubron on main road from Okaukuejo	
AF 259	6	Springbok	090302-01 ZH	Leeubron	
AF 260	6	Burchell’s zebr	090303-02 MK	NW of Okaukuejo, road to Leeubron	
AF 261	6	Springbok	090307-01 SB	Gravel Pit W of road to Leeubron	
AF 262	6	Springbok	090308-01 ZH	W of Leeubron	
AF 263	6	Burchell’s zebr	090311-01 MK	890 m NW of Leeubron, 70 m S of road	
AF 264	6	Burchell’s zebr	090312-01 MK	990 m SW of SA junction, 120 m S of road	
AF 310	6	Burchell’s zebr	090318-01 SB	SW of Leeubron	
AF 265	6	Burchell’s zebr	090324-01 ZH	W of Leeubron	
AF 311	6	Springbok	090326-01 MK	5.14 km NW of Okaukuejo	
AF 267	6	Burchell’s zebr	090401-01 MK	E of Sprokieswoud	
AF 270	6	Burchell’s zebr	090406-01 MK	~5 km NW of Okaukuejo; ~10.5 km SE of Leeubron	
AF 272	6	Burchell’s zebr	090407-01 ZH	Sprokieswoud-Adamax junction	
AF 274	6	Burchell’s zebr	090408-01 ZH	SA junction	
AF 278	6	Burchell’s zebr	090410-01 MK	~200 m N of E-W runway Okaukuejo airfield	
AF 279	6	Burchell’s zebr	090410-02 MK	Airfield	
AF 280	6	Burchell’s zebr	090411-01 SB	NW of SA (Sprokieswoud-Adamax) junction	
AF 288	6	Burchell’s zebr	090417-01 MK	Betsy's corner, road to Leeubron	probably the same outbreak, continuing
AF 289	6	Burchell’s zebr	090419 ZH	E of Leeubron	

AF 294	6	Burchell’s zebr	090423-01 MK	10 km NW of Okaukuejo on road to Leeubron	
AF 292	6	Springbok	090424-01 MK	4 km N of Okaukuejo	
AF 296	6	Springbok	090427-01 MK	3 km N of SA junction	
AF 300	6	Burchell’s zebr	090514-01 ZH	eastern bend in W drive	
AF 303	6	Burchell’s zebr	090526-01 ZH	~8 km S of Leeubron by trees in bend	
AF 305	6	Burchell’s zebr	090606-01 MT	~5 km NW of Okaukuejo	
AF 308	6	Burchell’s zebr	090623-01 SB	W of SA junction	
AF 336	6	Springbok	090703-01 SB	2k W of Leeubron	
AF 320	6	Burchell’s zebr	090707-01 SB	~300m S of gravel pit on way to Leeubron	
AF 334	6	Elephant	090716-01 MK	2.5km W of Aus	
AF 327	6	Burchell’s zebr	090825-01 ZH	~2 km NE of Okaukuejo, Rd to Gemsbokvlakte	
AF 323	6	Burchell’s zebr	090914-01 PK	just W of Gemsbokvlate, near junction N to main rd	
AF 322	6	Burchell’s zebr	090921-01 MK	~5 km SE of Leeubron	
AF 321	6	Burchell’s zebr	090929-01 MK	2.1 km S of Leeubron, SW of detour Rd	
AF 330	6	Burchell’s zebr	090929-01 WV	hill to NW of airfields	
AF 329	6	Burchell’s zebr	091001-01 HG	1.79 NNE of Okaukuejo	
AF 335	6	Burchell’s zebr	091115-01 MK	just S of Okaukuejo waterhole	
AF 318	6	Burchell’s zebr	091118-01 MK	4.6 km E of Sprokieswoud	
AF 319	6	Burchell’s zebr	091119-01 ZH	~ 2 km W of Leeubron	
AF 316	6	Burchell’s zebr	091120-01 ZH	turn-off to gravelpit S of Leeubron	

AF 353	6	Springbok	091201-01 MK	750 m NEN of Okaukuejo, rd to gravelpit by big Acacia	
AF 354	6	Burchell’s zebr	091203-01 MK	~3 km E of Kapupuhedi, 600m S of Road	
AF 252	6	Vulture	wingtag E025	Twin Trees, N of Okaukuejo	
AF 236	4	Burchell’s zebr	090103-01 BK	Okaukuejo airfield SE end of SE/NW strip	
AF 245	4	Springbok	090121-01 MK	~100 m W of junction two track to Grunewald	
AF 266	4	Burchell’s zebr	090327-01- MK	NW of Natco	
AF 287	4	Burchell’s zebr	090404-01TK	Fisher's pan, Namutoni	probably separate outbreak
AF 268	4	Burchell’s zebr	090405-01 MK	Grootvlakte, S Woud, ~150 m SW of horse camp	probably the same outbreak, continuing
AF 269	4	Burchell’s zebr	090405-02 MK	Gravel pit N of Grunewald	
AF 273	4	Burchell’s zebr	090407-02 ZH	Sprokieswoud-Adamax junction	
AF 275	4	Burchell’s zebr	090408-02 ZH	gravel pit by Adamax	
AF 276	4	Burchell’s zebr	090409-02 SB	West of Leeubron	
AF 277	4	Burchell’s zebr	090409-03 ZH	Okondeka-Adamax gravel pit	
AF 281	4	Burchell’s zebr	090411-01 ZH	SW of SA junction	
AF 282	4	Burchell’s zebr	090411-02 MK	SE of Grootvlatke horse camp	
AF 283	4	Burchell’s zebr	090414-01 MK	9 km N of Okaukuejo	
AF 284	4	Burchell’s zebr	090415-01 ZH	4 km NW of Okaukuejo	
AF 285	4	Burchell’s zebr	090416-01 ZH	Adamax-triangle, western 2 tracks	
AF 290	4	Burchell’s zebr	090421-01 SB	N side of western 2 track	

AF 295	4	Springbok	090424-01 SB	W of SA junction, N side of road	
AF 301	4	Springbok	090522-01 MK	2.39 km NE of Okaukuejo, S of west - east runway airfield	
AF 307	4	Burchell’s zebr	090616-01 JK	Nomab parking area in a road	
AF 328	4	Elephant	090716-01 MK	Gemsbokvlakte waterhole, 5 m E of trough	
AF 241	9	Springbok	090119-01 MK	Main road to Leeubron, near springbok stuck in a tree in October 2008	probably the same outbreak, continuing
AF 246	9	Springbok	090121-02 MK	NW of Okaukuejo	
AF 242	9	Blue wildebeest	090123-01 ZH	4.3 km W of Leeubron, ~70 m S of road	
AF 291	9	Burchell’s zebr	090422-01 MK	700 m S of Gemsbokvlakte at 10 m from road	recurrent or continuing outbreak
AF 297	9	Burchell’s zebr	090504-01 MK	~370 NW of Ombika waterhole (western detour)	
AF 299	9	Blue wildebeest	090507-01 MK	2.51 km NW of Okaukuejo	
AF 326	9	Burchell’s zebr	090716-01 MK	~3 km NW of Okaukuejo	
AF 333	9	Burchell’s zebr	090901-01 ZH	~1-2 km S of Okaukuejo, rd to Ombika	
AF 332	9	Burchell’s zebr	090914-01 MK	4.96 km NW of Okaukuejo	
AF 314	9	Gemsbok	SW 6318 I JB	Marula Game Ranch; Windhoek	not ENP
AF 317	9	Springbok	091119-01 WV	1.4 km N of Okaukuejo	recurrent or continuing from before
AF 239	5	Burchell’s zebr	090123 WT	S of Leeubron	
AF 304	5	Springbok	090604-01 RZ	Halali	probably separate outbreak

AF 254	5	Vulture	wingtag E025	Twin Trees, N of Okaukuejo	
AF 293	8	Burchell’s zebr	090424-01 MK	3.5 km NW of Okaukuejo	continuing outbreak
AF 302	8	Springbok	090522-02 MK	2.07 NE of Okaukuejo SW of N-runway	
AF 309	25	Springbok	090626-01 ZH	W-drive north of Gemsbokvlakte ~ 15 km	separate outbreak
AF 355	16	Burchell’s zebr	091213-01 BK	2km E of Okaukuejo, ~200 m S of main road	separate outbreak
AF 298	3	Burchell’s zebr	090505-01 MK	~800 m W of Okondeka, road to Adamax	separate outbreak
**YEAR 2010**					
AF 357	4	Burchell’s zebra	100201-02 SB	2 km north of twin trees	
AF 360	4	Burchell’s zebra	100202-01 MK	3.95 km north-west of Gemsbokvlakte, road between Gemsbokvlakte & main road Halali, 450 m east of road	
AF 359	4	Burchell’s zebra	100202-02 SB	~1 km south of Leeuboss	
AF 361	4	Burchell’s zebra	100202-02 MK	1.12 km north of Okaukuejo waterhole, 150 m east of road to airfield	
AF 362	4	Burchell’s zebra	100203-08 MK	5.23 km north-west of Okaukuejo, east of 2-track going south from main road to Leeubron	
AF 363	4	Burchell’s zebra	100203-07 MK	~3 km north-west of Okaukuejo, north of sewer road	

AF 365	4	Burchell's zebra	100203-04 MK	2.5 km north-west of Okaukuejo	
AF 369	4	Burchell's zebra	100204-03 SB	north of 2-track to Grunewald about 1 km west of main road to Leeubron	
AF 371	4	Burchell's zebra	100204-02 SB	300 m east of main road to Leeubron and 6.13 km north-west of Okaukuejo	
AF 372	4	Burchell's zebra	100205-01 ZH	east of main road near sewer road	
AF 375	4	Burchell's zebra	100208-02 SB	2.96 km north-west of Gemsbokvlakte, 100 m north of main road to Gemsbokvlakte	
AF 383	4	Burchell's zebra	100219-02 MK	1st gravel pit (100 m north-west of gravel pit) on way from airfield to Leeubron	
AF 386	4	Burchell's zebra	100219-01 SB	10.7 km north of airfield turnoff, 55 m east of road	
AF 382	4	Burchell's zebra	100219-01 MK	6.6 km north-west of Okaukuejo, road to Leeubron (after betsy's turn)	
AF 385	4	Burchell's zebra	100219-02 SB	1.24 km west of Okondeka 1 m north of road	
AF 388	4	Burchell's zebra	100222-04 SB	7.56 km south-west of Wolfnes, ~2 km east of Leeubron road & ~km west of Okondeka road	

AF 390	4	Blue wildebeest	100224-01SB	~70 m west of Okondeka road, ~300 m north of airfield turnoff	continuing outbreak
AF 392	4	Burchell's zebra	100224-02 WT	eastern 2-track in triangle, 100 m east of road	
AF 394	4	Burchell's zebra	100225-01 SB	~3 km south of Gaseb on west-drive, ~150 m west of road	
AF 395	4	Burchell's zebra	100228-01 MK	960 m north-east of Etosha Ecological Institute (EEI), ~400 m east of gravel pit	
AF 396	4	Burchell's zebra	100301-01 MK	2.73 km south-east of Adamax, 20 m off road eastside	
AF 397	4	Burchell's zebra	100301-01 SB	5.5 km west of Okaukuejo	
AF 398	4	Burchell's zebra	100302-01 SB	~3 km north of Natco, ~200 m west of road	
AF 404	4	Burchell's zebra	100317-02 SB	Okaukuejo waterhole	
AF 402	4	Burchell's zebra	100317-01 MK	4.52 km north-west of Okaukuejo, north of gravel pit across from north end of sewage 2-track	
AF 405	4	Burchell's zebra	100320-01 SB	5.41 km north of Okaukuejo, between Leeubron & Okondeka roads	
AF 407	4	Burchell's zebra	100321-01 SB	2.9 km north-north-east of Okaukuejo	
AF 409	4	Burchell's zebra	100323-02 MK	3.2 km north-west of Okaukuejo, road to Leeubron, 285 m east of sewage 2-track	

AF 410	4	Burchell's zebra	100323-01 SB	4.26 km north-west of Wolfnes, ~740 m north-west of Wolfsnes-Leeubron road	
AF 413	4	Burchell's zebra	100323-05 SB	512 m east of road, road to Leeubron, 6.91 km north-west of Okaukuejo	
AF 411	4	Burchell's zebra	100323-03 SB	4.8 km north-west of Leeubron, 458 m east of Leeubron 2-track	
AF 415	4	Burchell's zebra	100324-01 MK	2.5 km north of Okaukuejo, 1 km east of road to airfield	
AF 418	4	Burchell's zebra	100325-02 MK	723 m south of Okondeka, 160 m west of road	
AF 420	4	Burchell's zebra	100330-01 SB	400 m south of Sprokieswoud-Adamax junction	
AF 424	4	Burchell's zebra	100402-01 SB	1.24 km north of Natco, 180 m east of road	
AF 425	4	Burchell's zebra	100406-02 SB	7.45 km east of Adamax, 122 m north of road	
AF 356	6	Burchell's zebra	100115-01 MK	2.67 km east of Leeubron, south of Leeubron-Okondeka road	
AF 358	6	Springbok	100201-02 ZH	where western 2-track meets with Adamax-Okondeka road	
AF 364	6	Burchell's zebra	100203-05 MK	~4 km north-west of Okaukuejo off of sewer road	
AF 366	6	Burchell's zebra	100203-03 MK	~400 m north-east of East-West runway	

AF 367	6	Burchell's zebra	100203-02 MK	0.75 km north of East-West runway	
AF 368	6	Burchell's zebra	000203-01 MK	3.33 km north-west of Okaukuejo, ~50 m east of East-West runway	
AF 370	6	Burchell's zebra	100204-04 SB	north-east of Leeubron on road to Wolfsnes, south side of road	
AF 373	6	Burchell's zebra	100205-01 MK	3.22 km north-east of Okaukuejo, 980 south-east of East-West Runway	
AF 374	6	Springbok	100208-01 MK	600 m north of Airfield, 3.6 km north of Okaukuejo	
AF 376	6	Springbok	100209-01 MK	2.2 km north of exclosure (eastern 2-track)	continuing outbreak
AF 377	6	Burchell's zebra	100209-01 MK	3.62 km south-east of Leeubron, 600 m west of main road	
AF 378	6	Blue wildebeest	100212-01 SB	0.76 north-west of Gemsbokvlakte	
AF 379	6	Burchell's zebra	100215-01 MK	~4 km east of Sprokieswoud-Adamax junction	
AF 380	6	Burchell's zebra	100218-01 SB	~5 km west of Adamax on old track	
AF 384	6	Burchell's zebra	100219-02 WT	5.6 km north-west of Okaukuejo, ~200 m west of road to Leeubron, south of two track to Grunewald	

AF 389	6	Burchell's zebra	100222-01 SB	880 m east of road, 2.8 km south-west of Wolfsnes	
AF 393	6	Springbok	100223-01 WT	1 km north of Wolfsnes turnoff on side of road, 0.5 m off road	
AF 391	6	Burchell's zebra	100224-01 WT	370 m west of Leeubron detour, 630 m south of Leeubron-Sprokieswoud-Adamax road	
AF 399	6	Blue wildebeest	100301-01 BK	1 km from Namutoni towards Causeway	separate outbreak
AF 400	6	Burchell's zebra	100307-01 SB	~300 m south of main road, ~ 1 km east of Gemsbokvlakte turnoff (1st one)	
AF 403	6	Burchell's zebra	100317-01 SB	5.83 km north-north-west of Okaukuejo, 1.27 km west of road to Okondeka, west of first Acacia on road north	
AF 406	6	Burchell's zebra	100320-02 SB	5.04 km north of Okaukuejo, between Leeubron & Okondeka roads	
AF 414	6	Springbok	100323-02 SB	300 m north-west of Leeuboss	
AF 412	6	Burchell's zebra	100323-04 SB	4.45 km south-west of Leeubron, 1.21 km east of SA junction road	
AF 408	6	Burchell's zebra	100323-01 MK	3.2 km north of Okaukuejo, 420 m south of East-West Runway	continuing from before

AF 417	6	Blue wildebeest	100324-01MK	4.2 km north-north-east of Okaukuejo, 469 m north-north-east of East-West runway, 796 m south-west of North-South runway (North end)	from before
AF 419	6	Burchell’s zebra	100325-03 MK	890 m south-west of Okondeka, 608 m west of road	
AF 422	6	Burchell’s zebra	100331-01 SB	2.4 km west of Leeubron, 750 m north of road	
AF 421	6	Burchell’s zebra	100331-01 ZH	81 m east of Eastern-2-track (368 m north of Leeubron-Okondeka road)	
AF 423	6	Springbok	100402-01 SB	5.07 km east of Adamax, 155 m south of road	
AF 431	6	Springbok	100409-01 MK	2.1 km north of Okaukuejo road to airfield, 300 m east of road	
AF 416	8	Springbok	100324-01 MK	5 km south-west of Leeubron, 560 m west of road	separate outbreak
AF 387	9	Springbok	100220-01 MK	6.2 km north-west of Okaukuejo, ~250 m north of Grunewald two track off road to Leeubron	separate outbreak
AF 381	35	Burchell’s zebra	100219-01 WT	3.2 km east-north-east of Leeubron, 520 m south of road Leeubron-Okondeka	separate outbreak

AF 401	37	Burchell's zebra	100311-02 SB	2.1 km east of Okaukuejo, ~300 m north of Gemsbokvlakte road	separate outbreak
AF 434		Burchell's zebra	100221-02 SB	Okondeka road, about 6.7 km north of airfield turnoff, 20 m west of road	
AF 436		Burchell's zebra	100221-03 SB	7.98 km from Okaukuejo, on road to Leeubron, ~30 m west of road	
AF 437		Burchell's zebra	100301-01 ZH	Sprokieswoud, north side of road across from fenced area	
AF 435		Burchell's zebra	100306-01 SB	5.2 km north-north-east Natco, 195 m west of road	
AF 438		Burchell's zebra	100311-01 SB	1.4 km east of Okaukuejo	
AF 439		Burchell's zebra	100319-01 ZH	~4 km south of Leeubron, east side of road	
AF 442		Burchell's zebra	100319-02 ZH	Okaukuejo, ~1 km north of twin trees on west side of road	
AF 426		Burchell's zebra	100408-01 SB	450 m south of Okaukuejo waterhole, 140 m south of road to water tower	
AF 427		Burchell's zebra	100408-02 SB	7.09 km south-south-west of Leeubron	
AF 428		Burchell's zebra	100408-03 SB	6.53 km south-south-west of Leeubron	
AF 429		Burchell's zebra	100408-04 SB	8.03 km north-west of Okaukuejo	

AF 432		Burchell’s zebra	100409-03 SB	~2 km west-south-west of Leeubron, ~100 m south of road to Sprokieswoud-Adamax	
AF 430		Burchell’s zebra	100409-01 ZH	open plain between Leeubron & Sprokieswoud-Adamax junction, ~2 km west of Leeubron	
AF 443		Burchell’s zebra	100411-01 MK	2 km south-east of Sprokieswoud-Adamax junction, 360 m south off road	
AF 433		Burchell’s zebra	100411-02 MK	track to Adamax gravel pit, gravel pit 3 m west of road (350 m north of gravel pit)	
AF 444		Burchell’s zebra	100412-01 ZH	800 m east of Sprokieswoukd-Adamax junction	
AF 445		Burchell’s zebra	100413-01 SB	~500 m north of road, 3.06 km west of Sprokieswoud-Adamax junction	
AF 446		Blue wildebeest	100413-01 BK	~1 km east of Sprokieswoud, ~ 300 m north of road	
AF 447		Burchell’s zebra	100413-02 SB	~100 m north-east of Grunewald gravel pit	
AF 448		Black rhinoceros	100413-01 MK	832 m north-north-east of Homob Toilet camp, in gravel pit	
AF 451		Burchell’s zebra	100414-01 SB	7 km north of Okaukuejo, ~660 m west of Okondeka road	

AF 452		Burchell's zebra	100414-02 SB	6.21 km N of Leeubron, 245m W of Leeubron 2-track	
AF 450		Blue wildebeest	100414-01 SB	7.88 km south-east of Leeubron, 400 m south of road	
AF 449		OM	100414-01 SB	5.5 km north-west of Okaukuejo, 1.65 km east of road to Leeubron	
AF 453		Burchell's zebra	100414-04SB	1 km north-east of Sprokieswoud-Adamax junction, ~800 m east of road to Adamax	
AF 454		Springbok	100415-02 SB	1.79 km south of Sprokieswoud-Adamax	
AF 455		Burchell's zebra	100415-01 SB	3.51 km south-west of Leeubron, 1 km south of Sprokieswoud-Adamax, 641 m west of road	
AF 456		Burchell's zebra	100415-02 SB	1.19 km south-west of Sprokieswoud-Adamax, 872 m south of road	
AF 457		Burchell's zebra	100415-03 SB	1.92 km south of Sprokieswoud-Adamax, 1.11 km west of road	
AF 459		Burchell's zebra	100416-02 SB	~100 m north of Okondeka-Adamax road, ~9 km west of Okondeka	
AF 458		Burchell's zebra	100416-01 SB	1 km west of Adamax gravel pit, ~600 m south of Okondeka-Adamax road	

AF 461		Burchell's zebra	100421-02 SB	5.02 km south-west of Natco, 1.69 km south-west of road to Sprokieswoud-Adamax	
AF 460		Burchell's zebra	100421-01 SB	4.46 km north-west of Okaukuejo	
AF 463		Burchell's zebra	100423-02 ZH	north of Okaukuejo, 2 km from twin trees	
AF 462		Burchell's zebra	100423-01 ZH	~2 km south of Wolfsnes, ~80 m north of two Acacia on the road to Okondeka	
AF 464		Blue wildebeest	100424-01 ZH	north of Okaukuejo, ~2 km from Okaukuejo and about 800 m north of sewage turnoff	
AF 465		Burchell's zebra	100427-01 ZH	890 m north-west of Okaukuejo, west of road to sewage	
AF 467		Springbok	100428-01 MK	800 m east of Sprokieswoud, 80 m north of road	
AF 466		Blue wildebeest	100428-01 MK	5.1 km west of Okondeka, ~210 m north of road to Adamax	
AF 468		Burchell's zebra	100502-01 WB	8.2 km north-west of Okaukuejo, road to Okondeka, ~20 m west of road	
AF 470		Burchell's zebra	100503-03 MK	4.6 km south of Okaukuejo, Stark's pan, 130 m east of road	

AF 469		Burchell’s zebra	100503-01 MK	Okaukuejo-Okondeka-Leeubron junction	
AF 472		Springbok	100506-01	~4 km south of Leeubron and 100 m north of the vulture roosting trees	
AF 471		Springbok	100509-01 MK	410 m east of northern end of North-South-Runway	
AF 473		Burchell’s zebra	100510-01 MK	4.5 km west-north-west of Okaukuejo, 800 m north of two track to Grunewald	
AF 474		Burchell’s zebra	100525-01 CC	north-east of Gemsbokvlakte, 5 km from Gemsbokvlakte close to the end of transect 34	
AF 475		Burchell’s zebra	100531-01 MK	3.1 km south of Gaseb, west drive, east of road	
AF 476		Burchell’s zebra	100602-01 MK	15.4 km south-west of Gemsbokvlakte, south-east loop of west drive, 625 m west of road	
AF 477		Springbok	100603-01 MK	940 m south of Gaseb, 370 m west of West-Drive-Road	
AF 478		Burchell’s zebra	100603-01 MK	3.4 km south of Gemsbokvlakte, 550 m west of West-Drive	
AF 479		Burchell’s zebra	100607-02 MK	750 m south of Gemsbokvlakte, ~300 m east of road	

AF 480		Burchell's zebra	100607-03 MK	1.2 km south of Gemsbokvlakte, West-Drive, ~50 m west of road in Catophractes		
AF 481		Burchell's zebra	100607-04 MK	400 m south of Gemsbokvlakte, road to W-Drive, east of road 34 m		
AF 506		Elephant	100606-01 OA	Aus waterhole		
AF 505		Springbok	100628-01 MK	620 m north-east of Newbrownii, 200 m north of road		
AF 483		Burchell's zebra	100614-01 MK	Diamond drive, top intersection, 265 m south of main road to Newbrownii		
AF 496		Burchell's zebra	100617-01 MK	2.3 km north-west of Gemsbokvlakte		
AF 484		Burchell's zebra	100618-01 SB	2 km away from Okaukuejo on road to Gemsbokvlakte		
AF 485		Burchell's zebra	100621-01 ZH	W-Drive, east of Gaseb, ~4 km away		
AF 486		Burchell's zebra	100622-01 WV	Halali plains west		
AF 487		Burchell's zebra	1000625-01 SB	W-Drive ~1 km from Gemsbokvlakte, 50 m north of road		
AF 488		Burchell's zebra	100628-01 ZH	Gemsbokvlakte 200 m from waterhole		
AF 489		Burchell's zebra	100706-01 MK	1.7 km north-east of W-Drive gravel pit, 1 km east of W-Drive-Road		
AF 490		Burchell's zebra	100707-01 MK	1.6 km south of Gemsbokvlakte, 700 m east of road		

AF 491		Burchell’s zebra	100707-02 MK	1.2 km south of Gemsbokvlakte, ~80 m west of road, down W-Drive	
AF 494		Burchell’s zebra	100707-03 MK	990 m north of Gemsbokvlakte, road to main road, 220 m north of road	
AF 492		Springbok	100708-01 WV	2.8 km south of Gemsbokvlakte, 180 m east of road	
AF 493		Burchell’s zebra	100714-01 SB	3.37 km south of Gonob, 50 m east of track	
AF 495		Burchell’s zebra	100719-01 MK	~2.8 km east of Gaseb turn-off, road to Gemsbokvlakte, ~400 m north of road	
AF 497		Springbok	100723-01 MK	650 m south-east of Okaukuejo, south of location, 230 m east of road	
AF 498		Elephant	100726-01 ZH	700-800 m south of Okaukuejo, west of tar road	
AF 499		Springbok	100801-01 RZ	1.1 km north-north-west of Okaukuejo,~250 m north-east of gravelpit by big Acacia	
AF 500		Springbok	100811-01 MK	two-track to Grunewald	
AF 501		LA	100828-01 MK	Okaukuejo waterhole	
AF 502		Burchell’s zebra	100830-02 ZH	~4 km south-east of Gemsbokvlakte, W-Drive	
AF 503		Burchell’s zebra	100906-01 ZH	~150 m north of Okaukuejo waterhole	

AF 504		Elephant	100908-01 WK	700 m along eastern detour to Ombika, southern intersection	
not yet analyzed		Burchell's zebra	100916-01 WV	on powerline corner south of location??? ~700 m	
		Burchell's zebra	100922-01 WV	open plain between Okaukuejo and airfield	
		Gemsbok	100927-01 MK	3.16 km south of Okaukuejo, 650 m east of tar road	
		Blue wildebeest	100929-01 ZH	10 km south of Leeubron, south of road	
		Springbok	100930-01 ZH	~4 km south of Leeubron, west of road	
		Elephant	100930-01 ZH	100-150 m north of Duiwelsvuur	
		Elephant	100930-02 ZH	200 m east of Duiwelsvuur waterhole	
		Black rhinoceros	101012-01 BK	Nerens	
		Burchell's zebra	101012-01 MK	840 m south-south-west of Okaukuejo, south of 2-track	
		Elephant	101012-01 MK	4.64 km south-east of M'Bari, ~15m south of road	
		Giraffe	101013-01 MK	~600 m north of Okondeka waterhole	
		Burchell's zebra	101019-01 CC	3.85 km north-east of Gemsbokvlakte, road to Nebrownii	
		Burchell's zebra	101019-01 MK	4.5 km north-north-east of Okaukuejo, east of East-West runway	

		Burchell's zebra	101019-02 MK	4.3 km north-north-east of Okaukuejo, edge of pan	
		Burchell's zebra	101019-04 MK	just east of Gemsbokvlakte	
		Burchell's zebra	101019-05 MK	~6 km north of Okaukuejo, road to Okondeka, 50 m west of road	
		Burchell's zebra	101021-01 ZH	just east of Gemsbokvlakte	
		Springbok	101025-01 MK	580 m south-south-west of Gemsbokvlakte, W-drive	
		Burchell's zebra	101112-01 ZH	4.9 km north-east?? of Okaukuejo, Leeubron road	
		Black rhinoceros	101114-01 CC	1.5 km south of Gemsbokvlakte, W-drive	
